# Machine Learning-Enhanced Flexible Mechanical Sensing

**DOI:** 10.1007/s40820-023-01013-9

**Published:** 2023-02-17

**Authors:** Yuejiao Wang, Mukhtar Lawan Adam, Yunlong Zhao, Weihao Zheng, Libo Gao, Zongyou Yin, Haitao Zhao

**Affiliations:** 1 Applied Mechanics Laboratory, Department of Engineering Mechanics, Tsinghua University, Beijing, 100084 People’s Republic of China; 2 Materials Interfaces Center, Shenzhen Institute of Advanced Technology, Chinese Academy of Sciences, Shenzhen, 518055 People’s Republic of China; 3 Department of Mechanical and Electrical Engineering, Xiamen University, Xiamen, 361102 People’s Republic of China; 4 School of Mechano-Electronic Engineering, Xidian University, Xi’an , 710071 People’s Republic of China; 5 Research School of Chemistry, Australian National University, Canberra, ACT 2601 Australia

**Keywords:** Flexible mechanical sensors, Machine learning, Artificial intelligence, Data processing

## Abstract

To realize a hyperconnected smart society with high productivity, advances in flexible sensing technology are highly needed. Nowadays, flexible sensing technology has witnessed improvements in both the hardware performances of sensor devices and the data processing capabilities of the device’s software. Significant research efforts have been devoted to improving materials, sensing mechanism, and configurations of flexible sensing systems in a quest to fulfill the requirements of future technology. Meanwhile, advanced data analysis methods are being developed to extract useful information from increasingly complicated data collected by a single sensor or network of sensors. Machine learning (ML) as an important branch of artificial intelligence can efficiently handle such complex data, which can be multi-dimensional and multi-faceted, thus providing a powerful tool for easy interpretation of sensing data. In this review, the fundamental working mechanisms and common types of flexible mechanical sensors are firstly presented. Then how ML-assisted data interpretation improves the applications of flexible mechanical sensors and other closely-related sensors in various areas is elaborated, which includes health monitoring, human–machine interfaces, object/surface recognition, pressure prediction, and human posture/motion identification. Finally, the advantages, challenges, and future perspectives associated with the fusion of flexible mechanical sensing technology and ML algorithms are discussed. These will give significant insights to enable the advancement of next-generation artificial flexible mechanical sensing.

## Highlights

- The latest progress on the integration of flexible mechanical sensing platforms with machine learning (ML) is reviewed.
- The advantages, challenges, and future perspectives of the application of ML to intelligent flexible mechanical sensing technology are discussed.
- The fundamental working mechanisms and common types of flexible mechanical sensors are reviewed.

## Graphical abstract

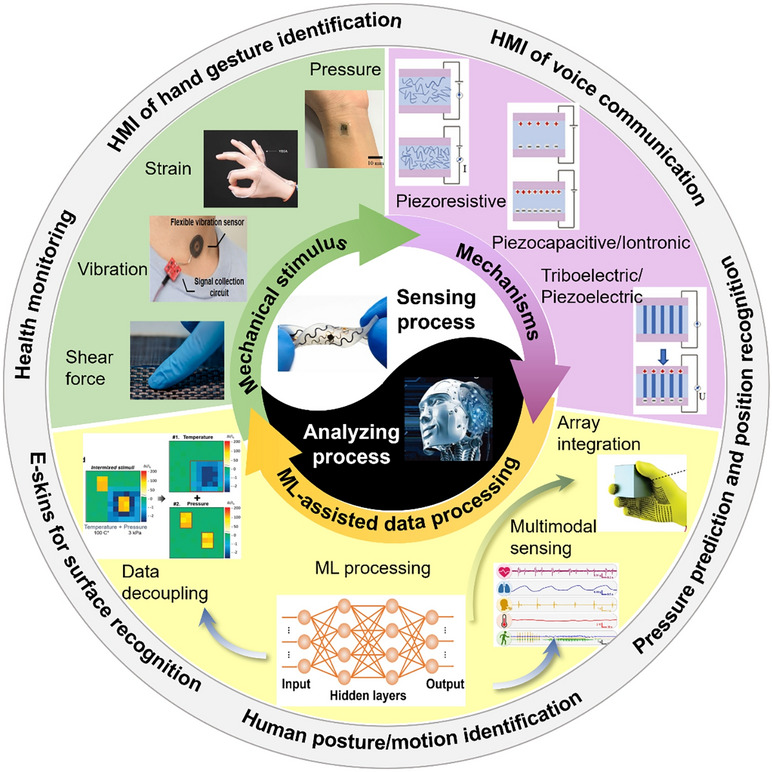

## Introduction

**Fig. 1:**
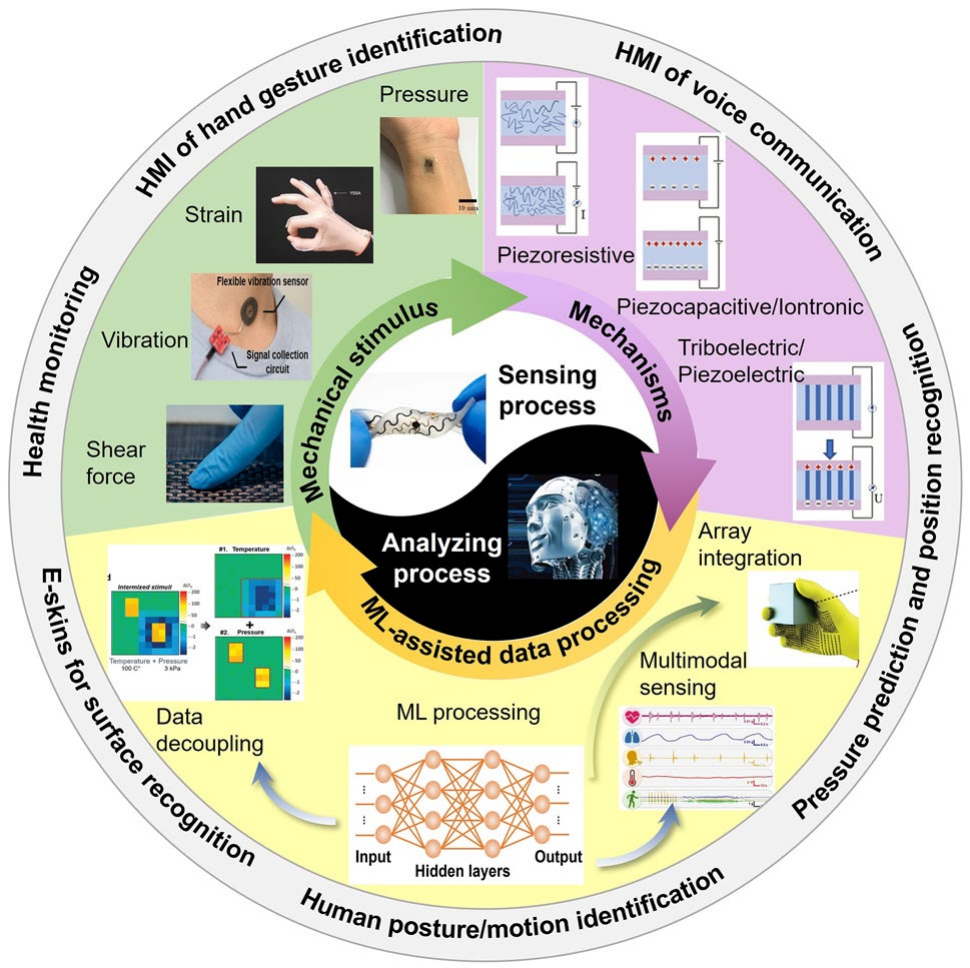
An overview of stimuli, mechanisms, and ML-assisted data processing of flexible mechanical sensing technology. Mechanical stimuli: pressure [15] (Copyright (2019) The Authors), strain [23] (Copyright (2020) Springer Nature), vibration [31] (Copyright (2022) The Authors), and shear force [32] (Copyright (2020) American Chemical Society). Mechanisms: piezoresistive, piezocapacitive/iontronic, and triboelectric/piezoelectric [33] (Copyright (2021) Wiley–VCH). ML-assisted data processing: array integration [34] (Copyright (2019) Springer Nature), multimodal sensing [35] (Copyright (2020) The Authors), and data decoupling [36] (Copyright (2020) Wiley–VCH). Sensing process [37] (Copyright (2022) Elsevier) and analyzing process [20] (Copyright (2021) Wiley–VCH)

In the new era of smart society, flexible electronics with various functionalities have experienced bloom developments with the rapid progress of the Artificial Intelligence of Things (AIoT) and fifth-generation (5G) communication technology [1–4]. Flexible and stretchable mechanical sensors as one important part are attracting extensive research as they possess the capability to quantify external mechanical stimuli such as pressure, strain, shear force, and vibration, via electrical signals [5–8] (Fig. 1). Compared to traditional rigid sensors, flexible mechanical sensors can even be deformed into any shapes to conform with the surface of human skin, robotic/prosthesis, and smart devices, endowing them with smart sensing abilities. Meanwhile, flexible mechanical sensors have also been developed with other novel characteristics, notably optical transparence to function in a visually imperceptible manner [9–12]. Hence, a wide range of applications have been established, such as health/motion monitoring [13–17], human machine interface (HMI) [18–20], and smart home [1, 21], among others.

As fundamentals, different sensing mechanisms have been developed from piezoresistive and capacitive sensors with high sensitivities, to piezoelectric and triboelectric sensors with a distinct advantage of zero power consumption. Based on these mechanical sensors, different physical parameters are recorded to perceive external stimuli with different sensing properties such as sensitivities, working range, linearity, and robustness, thus can be utilized in different scenarios. Moreover, to accomplish an intelligent sensing system that can not only detect but also analyze and make decisions, advanced data processing methods are correspondingly fused with flexible mechanical sensing technology. In particular, ML algorithms have been widely reported to conduct a more complicated and comprehensive analysis of the collected raw data of flexible sensors to effectively extract useful information [19, 22–24], far beyond the interpretability of conventional approaches. The trained models in ML have been used to classify, identify and predict values based on the designated tasks of the single sensor or multiple/multimodal sensors in the target application.

Lately, several reviews have covered related topics on the integration of flexible mechanical sensors with ML. However, most are dedicated to specific sensor types or applications such as self-powered mechanical sensors [25], stretchable sensors [26], piezoelectric acoustic sensors [27], flexible sensors for heart monitoring [28], and tactile sensors for HMI [29] and soft robots [30]. This review is aimed at providing a comprehensive survey of the common flexible mechanical sensor types and how the emerging ML algorithms impact the broad applications of flexible mechanical sensing. Firstly, we introduce various sensing mechanisms of flexible mechanical sensors, including both the conventional and recently emerging ones (Table 1) with representative calculation formulas to uncover the underlying physical changes. Then, the common mechanical sensor types to perceive pressure, strain, vibration, and shear stress with their main applications and typical design strategies are presented. Thirdly, how ML-assisted data analyzing methods contribute to various applications of flexible mechanical sensing technology is elaborated, including health monitoring, HMI, object/surface recognition, pressure prediction, and human posture/motion identification. Lastly, we summarize the advantages and challenges of integrating flexible mechanical sensing technology with ML algorithms, to promote the advancement of intelligent flexible mechanical sensing and other closely-related applications.

**Table 1:** Summary of piezoresistive, piezocapacitive, piezoelectric, iontronic, triboelectric, and piezoelectric sensing mechanisms of flexible mechanical sensors

Mechanisms	Advantages	Disadvantages
Piezoresistive	Easy to be integrated; Work well in array configuration; Simple device structures; Wide pressure sensing ranges; Easy fabrication processes	Hysteresis; Sensitive to temperature
Piezocapacitive	Low power consumption; Good dynamic response; Insensitive to temperature and humidity; Excellent proximity sensing ability under non-contact situations	Vulnerable to electromagnetic noises; Parasitic coupling with the surroundings; Complicated data measurement and processing
Iontronic	Ultra-high sensitivity; High noise immunity; High resolution and spatial definition; Suitable for static and dynamic stimuli	Inferior electrochemical stability of iontronic materials; Limited material longevity
Triboelectric	Self-powered; Great dynamic force-sensing ability; High power output; Non-contact sensing ability; Wide selection of materials	Unsuitable for static mechanical loads
Piezoelectric	Self-powered; Great dynamic force-sensing ability; Fast response time; Excellent high-frequency response for vibration measurements	Unsuitable for static mechanical loads; Require polarization process for many piezoelectric materials

## Working Mechanisms

### Piezoresistive Effect

Flexible piezoresistive sensors are built on the piezoresistive effect, which refers to the phenomenon that the resistance of material will change due to the variation of material geometry or resistivity when the material is loaded [38–40]. Since resistance change can be easily measured, flexible piezoresistive sensors gain great popularity and it is easy to integrate them into flexible electronic systems. These sensors also work well in array configuration since there is less crosstalk among adjacent units. In addition, they excel in simple device structures, wide pressure sensing ranges, and easy fabrication processes. But several drawbacks of these sensors should not be ignored including hysteresis and temperature sensitivity [41–44]. The underlying mechanisms of the piezoresistive effect can be induced by both the piezoresistive effect of the intrinsic materials and the resistance change caused by structures, as described in the following sections.

#### Piezoresistive Effect in Metal Conductors

Although most flexible piezoresistive sensors are realized through heterogeneous conductive media for large deformability and piezoresistivity [46], here we start with the piezoresistive effect in metal conductors and semiconductors to understand the basic mechanisms. First, let’s take a metal conductor with a cylindrical bar shape as an example [39]. While an electric field $\varepsilon$ is applied longitudinally to this conductor, its isotropic electrical resistance *R* can be determined by the resistivity $\rho$, the length *L*, and the cross-sectional area *A* as:

1 $$R=\frac{\rho L}{A}$$

Once the conductor is mechanically deformed under an applied tensile force, its resistance changes as:

2 $$\mathrm{d}R=\frac{\partial R}{\partial \rho }\mathrm{d}\rho +\frac{\partial R}{\partial l}\mathrm{d}l+\frac{\partial R}{\partial A}\mathrm{d}A$$

Then dividing by *R* and considering Poisson’s ratio *ν* will produce:

3 $$\frac{\mathrm{d}R}{R}=\frac{\mathrm{d}\rho }{\rho }+\frac{\mathrm{d}l}{l}(1+2v)$$

Hence, the longitudinal gauge factor $G{F}_{l}$, which represents the ratio of the change of $R$ to the change of $l$, can be calculated:

4 $$G{F}_{l}=\frac{\mathrm{d}R/R}{\mathrm{d}l/l}=1+2\upsilon +\frac{1}{{\varepsilon }_{l}}\frac{\mathrm{d}\rho }{\rho }$$

#### Piezoresistive Effect in Semiconductors

The piezoresistive effect in semiconductors is generally much stronger than that in metals [40]. Different from purely geometrical influence, the piezoresistive effect in semiconductors appears at the atomic level [47]. Although it is less evident than the former, it also contributes to the strain dependence of resistance changes. Thus, typical elastic semiconductor materials, such as silicon and gallium arsenide, present obvious differences in the gauge factor calculated by Eq. (4). The conductivity change is caused by the change of concentration (*n*) and mobility (*μ*) of free electrons due to the lattice deformation. The doping type, level, and crystallographic direction may strongly affect the gauge factors along the longitudinal and transverse directions. The resistivity of the materials (*ρ*) can be calculated by:

5 $$\rho =\frac{1}{ne\mu }$$

where *e* is the electron charge. Substituting in Eq. (4), we obtain the gauge factor

6 $$GF=1+2\upsilon -\frac{1}{\varepsilon }\frac{\mathrm{d}(n\mu )}{n\mu }$$

#### Piezoresistive Effect in Polymer Composites

**Fig. 2:**
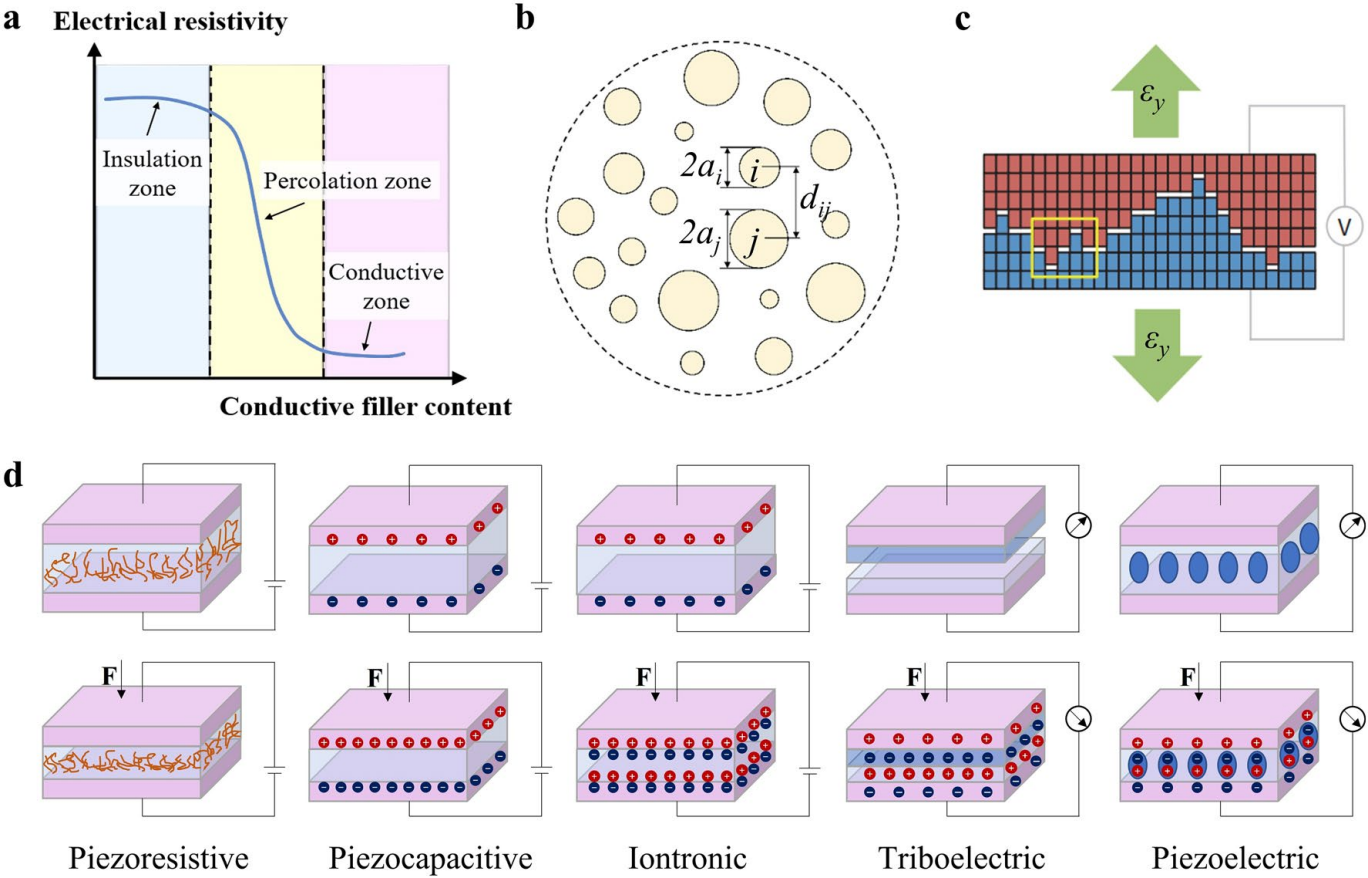
Mechanical sensing mechanisms. **a** Percolation theory for conductive filler-doped polymer composites. **b** Contact resistance change analysis for a simplified case of a set of circular contact spots. **c** Crack-induced resistance change analysis for a transverse crack with nanoscale zigzag edges [45]. Copyright (2014) Springer Nature. **d** Schematic diagrams for five common sensing mechanisms

As one of the mostly studied sensing materials for flexible piezoresistive mechanical sensors, conductive filler-doped polymer composites present a conduction mechanism that can be explained by percolation theory with a phenomenological description [48, 49]. Briefly, conductive fillers, like metal nanoproducts (e.g., Ag/Cu nanowires [50–52]) and carbon-based fillers (e.g., graphene [53], carbon nanotube [54], carbon black [55]), are quite isolated at low filler concentrations in the polymer matrix, resulting in high electrical resistances. As the concentration increases to a point, a conductive path spanning the whole polymer system is formed due to the contact of conductive fillers, along with a drastic increase in electrical conductivity (Fig. 2a). This critical point is defined as the “percolation threshold”, *P*_*c*_. The conductivity $\sigma$ of composite polymer above the percolation threshold follows [56]:

7 $$\sigma ={{\sigma }_{0}\left(P-{P}_{c}\right)}^{n}$$

where *P* represents the volume fraction of the filler, and *n* is the power of conductivity increase after achieving the percolation threshold. The value of *n* is largely decided by the filler properties [57]. Once the composite conductors are mechanically deformed, the volume fraction of the filler changes, thus leading to a change in conductivities.

#### Contact Resistance Change

In addition to the interior resistance variation of sensing materials, the piezoresistive response of flexible mechanical sensors can also be induced by the change in contact resistance between the electrode and sensing material or two electrode sheets (electrodes serving as sensitive elements in some sensors). Various surface/porous microstructures have been developed to take advantage of the contact resistance change to increase the sensitivities [58–63]. Usually, it is difficult to calculate the contact resistance considering the practically rough surface with numerous spots to form conducting paths of electrons. Here we refer to J. A. Greenwood’s theory which simplifies the situation by considering the cluster of perfect circular microcontacts (Fig. 2b) [64]. Based on this theory, extensive studies dealing with various electrical contacts have been conducted. The constriction resistance is expressed:

8 $$\begin{array}{c}\begin{array}{c}{R}_{G1}={R}_{{\text{par.}}}+{R}_{{\text{int.}}}=\frac{\rho }{2\sum {a}_{i}}+\frac{\frac{\rho }{\pi }\left(\sum_{i\ne j} \sum \frac{{a}_{i}{a}_{j}}{{d}_{ij}}\right)}{{\left(\sum {a}_{i}\right)}^{2}}\\ \end{array}\end{array}$$

where *R*_par._ and *R*_int._ represent the electrical resistance of all spots in parallel and the interaction among them. ${a}_{i}$ denotes the radius of the spot $i$, and ${d}_{ij}$ denotes the center distance between the spots $i$ and $j$. The formula can be further approximated when the n spots have the same size:

9 $$\begin{array}{c}{R}_{G2}\approx \frac{\rho }{2\sum {a}_{i}}+\frac{\rho }{\pi {n}^{2}}\sum_{i\ne j} \sum \frac{1}{{d}_{ij}}\end{array}$$

#### Crack-Induced Resistance Change

Constructing micro/nanoscale cracks of conductive films deposited on a soft substrate has also been proved to be an effective route to improve the piezoresistive response. A wide variety of conductive materials have been investigated in this strategy including metal nanofilms [45, 65, 66], metal nanowires/nanoparticles [67, 68], carbon-based materials [69–71], and conductive polymers [72]. During the deformation of the crack-based flexible mechanical sensors, these cracks of conductive films experience disconnection and reconnection with adjacent parts, changing the electrical current paths. Both cut-through [69–71] and non-through cracks [67, 72] have been exploited in flexible mechanical sensors. For a transverse crack with nanoscale zigzag edges (Fig. 2c) [45], the normalized crack conductance is calculated by

10 $$S=\frac{\sum_{i} {N}_{i}\theta \left({\varepsilon }_{i}-\varepsilon \right)}{\sum_{i} {N}_{i}}$$

where $\varepsilon$ denotes averaged crack gap displacement and ${N}_{i}$ denotes the number of crack asperity height ${\varepsilon }_{i}$.$\theta$ is the Heaviside step function. For conductive films with multiple similar transverse cracks, it can be inferred that the crack-induced resistance (*R* = 1/*S*) change is in proportion to the crack density with other parameters fixed.

### Piezocapacitive Effect

Flexible piezocapacitive mechanical sensors record capacitance change caused by deformation/deflection of a component electrode under the applied mechanical stimulus, resulting in the separation gap change of the capacitor sensor. The capacitive sensing mechanism endows these flexible sensors with the advantage of low power consumption, good dynamic response, and low susceptibility to temperature and humidity change compared to piezoresistive sensors. Uniquely, some piezocapacitive sensors present excellent proximity sensing ability under non-contact situations [73–76]. However, they are vulnerable to electromagnetic noises and the parasitic coupling with the surroundings needs to be carefully investigated and addressed, making it difficult in measuring and processing the capacitance data, especially in an array configuration. The piezocapacitive sensing devices are usually made up of two conductive parallel electrodes, separated by an insulating medium. Suppose $A$, $d$, and $\varepsilon$ representing the effective overlapping area, separation distance, and permittivity of the medium, respectively, the capacitance of the device is calculated by:

11 $$C=\frac{\varepsilon A}{d}$$

### Iontronic Sensing

A brand-new mechanical sensing mechanism known as iontronic sensing has been developed over the past decade [77–81]. Although similar to piezocapacitive sensors in signal sources to record capacitance change, iontronic sensors are built on the electrolytic-electronic interface where the electrical double layer (EDL) forms with the super capacitive nature, thus causing ultrahigh sensitivity and high noise immunity for the pressure sensing technology. The significant capacitance change is attributed to both the EDL formation and the change of contact area as the pressure is increased. The unique sensing mechanism with excellent properties also enables high resolution and spatial definition, and perception for both static and dynamic stimuli, via thin and flexible device architectures. However, the electrochemical stability of iontronic materials to resist temperature and humidity variations and the limited material longevity of the device constructs need to be resolved. Based on the deformation mode to increase the interfacial area, the existing iontronic pressure sensors can be divided into the following categories: (1) bending-dominated model; (2) elasticity-dominated model, and (3) combination of the first two [82].

An ionic material in film format is a common example of the structural bending model. The film iontronic sensor consists of a built-in spacer layer, which separates the electrode film and ionic film under a threshold pressure. As the applied pressure exceeds it, the two functional films come into contact and then the contact region between them continues to expand. The thin-plate deformation theory can be adopted to analyze the mechanical behaviors of the electrode film within the small deflection limit. Assuming that the top electrode contacts with the ionic film under applied pressure (*P*), the equivalent bending plate can be taken as a plate with a decreased surface area. Thus, the EDL capacitance of an iontronic sensor with a square membrane (*C*_EDL_) can be determined [83]:

12 $$ C_{{{\text{EDL}}}}  = {\text{UAC}} \cdot a\left( {a - \sqrt[4]{{\frac{{hD}}{{{\text{NP}}}}}}} \right) $$

where UAC denotes unit area capacitance; $a$ and $h$ represent the side length of the film sensor and the spacer’s height, respectively. $D$ and $N$ stand for the flexural rigidity and a geometrical constant of the boundary conditions of the top electrode membrane.

### Triboelectric Effect

Noticeably, most of the above mechanical sensors require an externally supplied power source, which largely limits their practical applications. Alternatively, this problem can be solved by utilizing the conversion of mechanical to electrical energy, and the corresponding sensors based on two mechanisms, namely triboelectric and piezoelectric effects, have been developed. For the triboelectric sensors, triboelectric charges are produced due to the coupling effect of contact electrification and electrostatic induction when two different materials come into frictional contact [84–86]. Triboelectric nanogenerators (TENGs) have been widely investigated for self-powered flexible mechanical sensors, in which the generated electrical output signal is influenced by both the magnitude and frequency of the external mechanical stimuli. Therefore, these sensors are mostly suitable for dynamic force sensing and can hardly detect static mechanical loads as they transfer charges only during the contact and release of two different materials with opposite charges. Triboelectric sensors have high power output even at low-frequency mechanical stimuli, non-contact sensing ability, and a wide material selection since the triboelectric effect occurs in various materials.

Two basic modes among others have been applied to flexible mechanical sensors, namely contact-separation mode and contact-sliding mode. For a device structure based on the first mode with two material layers as the metal–insulator triboelectric pair, the external pressure value is detected via the open-circuit voltage and the transferred charge density, whereas the rate of the pressure being applied is monitored by the pulse-like short-circuit current peak [87]. Under the open-circuit condition, the voltage ${V}_{OC}$ increases linearly as the applied pressure is withdrawn since the vertical gap distance between them ($d$) increases:

13 $$\begin{array}{c}{V}_{OC}=\frac{\sigma   \cdot   d}{{\varepsilon }_{0}} \end{array}$$

where $\sigma$, ${\varepsilon }_{0}$ represent the triboelectric charge density and permittivity of vacuum. Considering the materials resilience of the triboelectric pressure sensor as the spring-entangled structure with a material elastic modulus of $k$, the relationship between the applied pressure and $d$ is:

14 $$\begin{array}{c}p=\frac{k  \cdot   x}{S}=\frac{k\bullet \left({d}_{0}-d\right)}{S}\end{array}$$

Thus, we can obtain the change of open-circuit voltage decided by the magnitude of the applied pressure:

15 $$\frac{{V}_{OC,0}-{V}_{OC}}{{V}_{OC,0}}=\frac{{d}_{0}-d}{{d}_{0}}=\frac{S}{k\cdot {d}_{0}}\cdot p$$

### Piezoelectric Effect

The piezoelectric effect that has been applied in piezoelectric mechanical sensors is the direct piezoelectric effect, which defines the phenomenon whereby the deformation of certain dielectrics under an external force induces charge accumulation and thus voltage on two sides of the dielectric. The principle of the direct piezoelectric effect is illustrated in Fig. 2d. Once a tensile/compression external force is applied to a crystal in a certain direction, electric polarization occurs inside the crystal, and results in electric charges of + and − signs at its two surfaces simultaneously [88]. Reversibly, the removal of the applied external force automatically restores the crystal to its uncharged state. The direction of the electric charge polarity aligns with the applied external force, and the generated charge amount of the crystal is proportionally decided by the magnitude of the external force. Similar to triboelectric sensors, piezoelectric sensors are also only suitable for dynamic force sensing and not applicable to static mechanical loads due to the underlying variation-dependent sensing mechanism. Due to the instantaneous formation of a piezoelectric potential upon deformation, these sensors exhibit a fast response time and an excellent high-frequency response for vibration measurements. But it is necessary to conduct a polarization process for many piezoelectric materials to induce their piezoelectric response by applying DC voltage for modifying the randomly oriented domains [89, 90].

## Mechanical Sensing Types

### Flexible Pressure Sensors

**Fig. 3:**
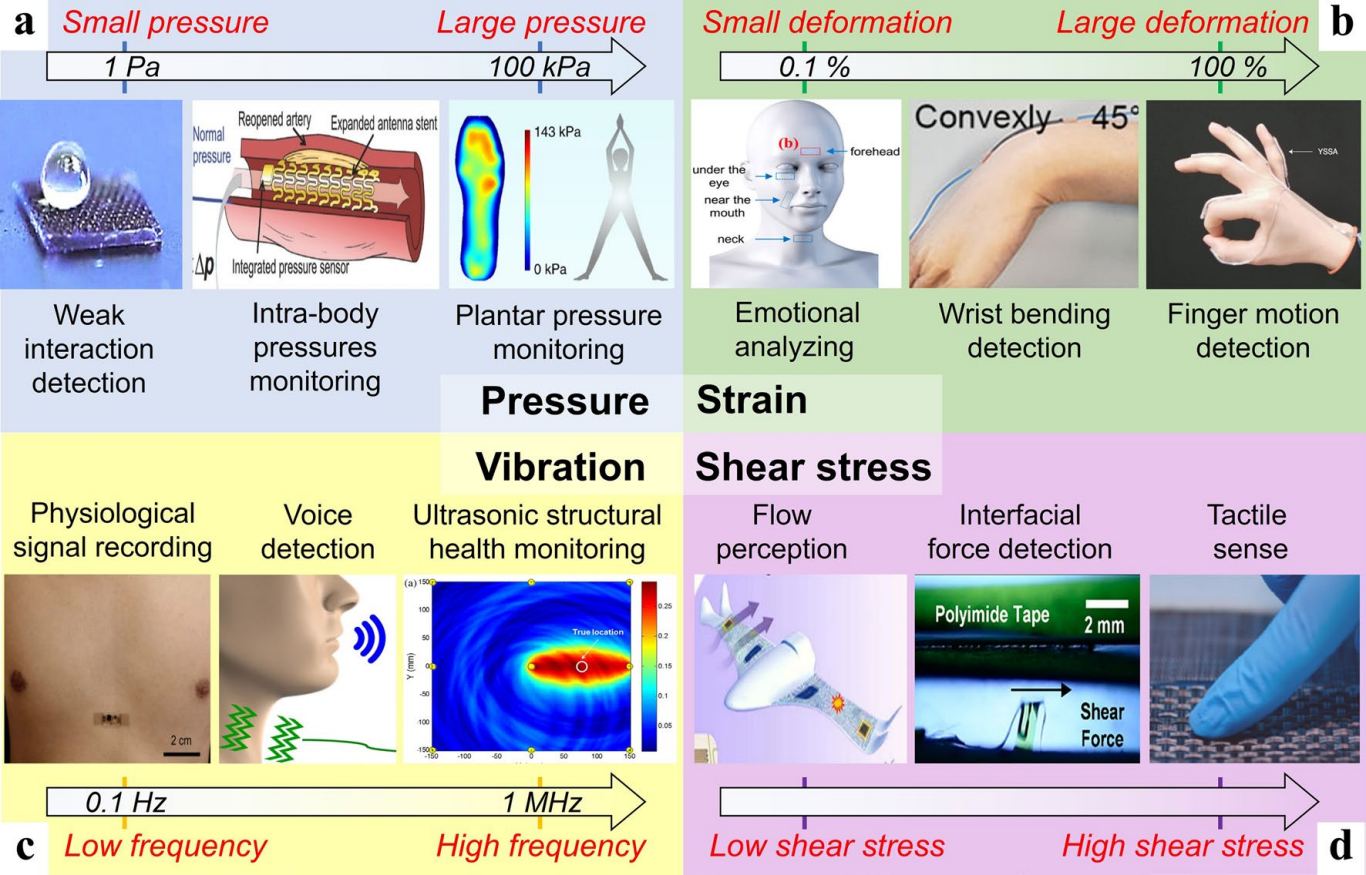
Common applications of four types of flexible mechanical sensing including **a** pressure sensing [91–93] (Copyright (2017) The Authors, Copyright (2020) The Authors, Copyright (2020) The Authors), **b** strain sensing [23, 94, 95] (Copyright (2015) American Chemical Society, Copyright (2020) Springer Nature, Copyright (2020) Wiley–VCH), **c** vibration sensing [96–98] (Copyright (2017) Elsevier, Copyright (2019) The Authors, Copyright (2016) The Authors), and **d** shear stress sensing [32, 99, 100] (Copyright (2021) Elsevier, Copyright (2019) American Chemical Society, Copyright (2020) American Chemical Society)

Pressure is one of the most common mechanical stimuli that need to be sensed in nature and humans. A large community of flexible pressure sensors has been developed as the detection of pressures ranging from several pascals to hundreds of kilopascals is required for various applications (Fig. 3a).

**Fig. 4:**
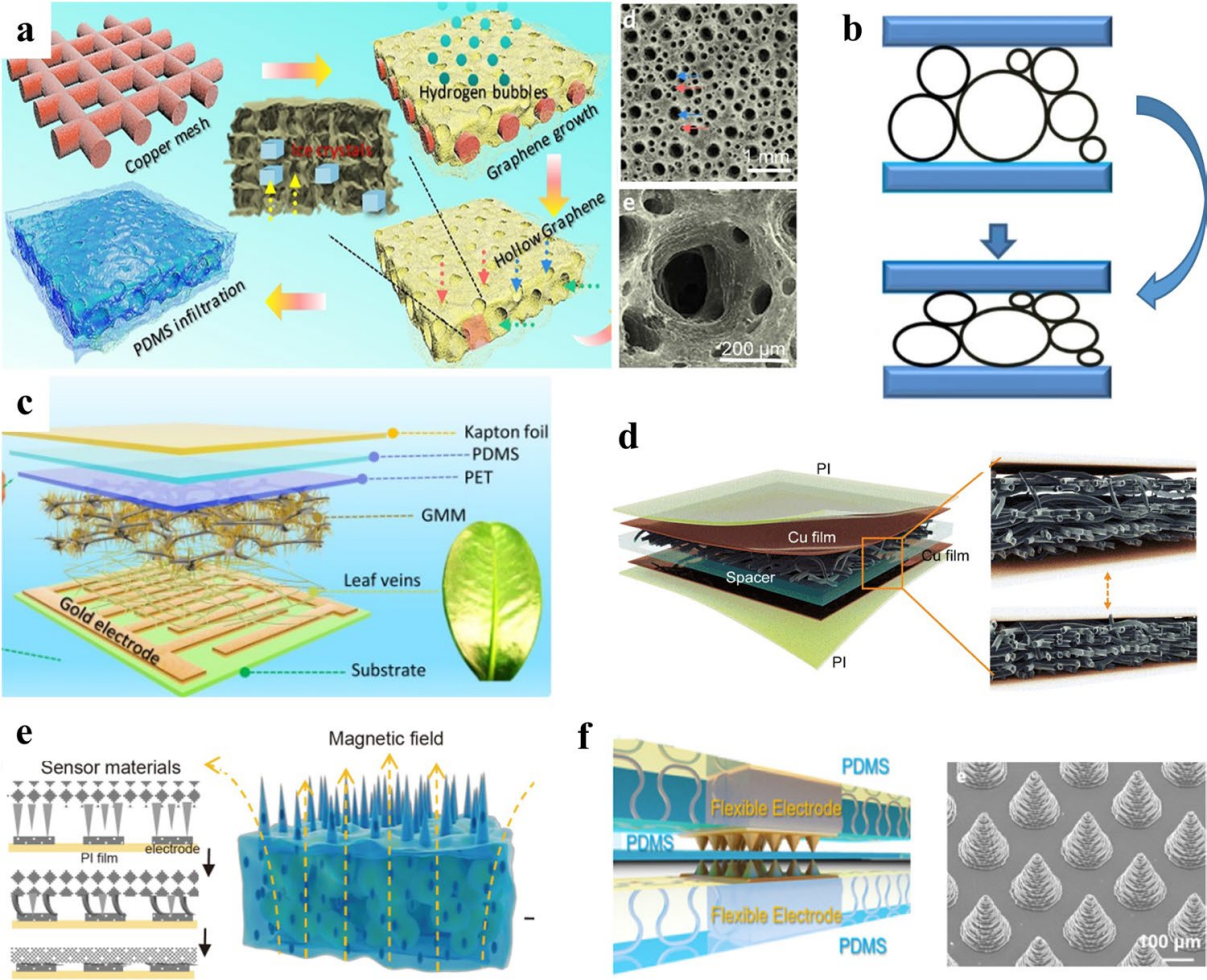
Typical design strategies of flexible pressure sensors. **a** Hierarchically porous graphene structures [107]. Copyright (2019) American Chemical Society. **b** Conducting polymer films with hollow-sphere microstructures [106]. Copyright (2014) Springer Nature. **c** A melamine foam as the flexible scaffold of sensing materials [108]. Copyright (2020) Elsevier. **d** Cross-interlocked nylon fabrics coated by PEDOT:PSS [109]. Copyright (2022) IEEE. **e** Porous elastomer with surface micropillar arrays [110]. Copyright (2021) Springer Nature. **f** Conical surface microstructures on sensor electrodes [111]. Copyright (2021) American Chemical Society

Commonly measurable pressures can be divided into four ranges including subtle-pressure (1–1 kPa), low-pressure (1–10 kPa), medium-pressure (10–100 kPa), and high-pressure regimes (> 100 kPa). The subtle-pressure regime (1–1 kPa) covers pressures created by weak interaction and many small object weights. For example, putting a pencil on a flat surface causes a pressure of around 300 Pa and a layer of pencil shavings induces a pressure of about 40 Pa [80], and a water droplet generates a pressure of about 13 Pa [101]. A sensitive response in the subtle-pressure region is critical for developing pressure sensors assembled in highly sensitive touch screen devices. Many pressures induced by gentle manipulation of items and intra-body pressures of humans (e.g., intraocular pressure and intracranial pressure) usually fall within the low-pressure regime (1–10 kPa). Pressure sensors showing excellent performance in this regime are receiving considerable attention for applications in e-skin and health monitoring/diagnosis systems [101–103]. The medium-pressure regime (10–100 kPa) concludes atmospheric pressure at high altitudes and average plantar pressure during standing still. A higher value of plantar pressure distribution during human motions can easily exceed this range and thus flexible pressure sensors still working well when reaching the high-pressure regime (> 100 kPa) are preferred in monitoring the plantar pressure for motion and gait analysis [91, 104, 105]. Typical design strategies of flexible pressure sensors include exploiting porous structures (Fig. 4a-d) and surface microstructures (Fig. 4e, f) to perceive pressure stimuli with high sensitivities. The hollow-sphere microstructure of conducting polymer thin film in Fig. 4b was developed by Bao’s group to realize an unprecedented sensitivity of 133 kPa^−1^ along with a low detection limit of 0.8 Pa, surpassing the subtle-pressure-sensing properties of human skin [106].

### Flexible Strain Sensors

**Fig. 5:**
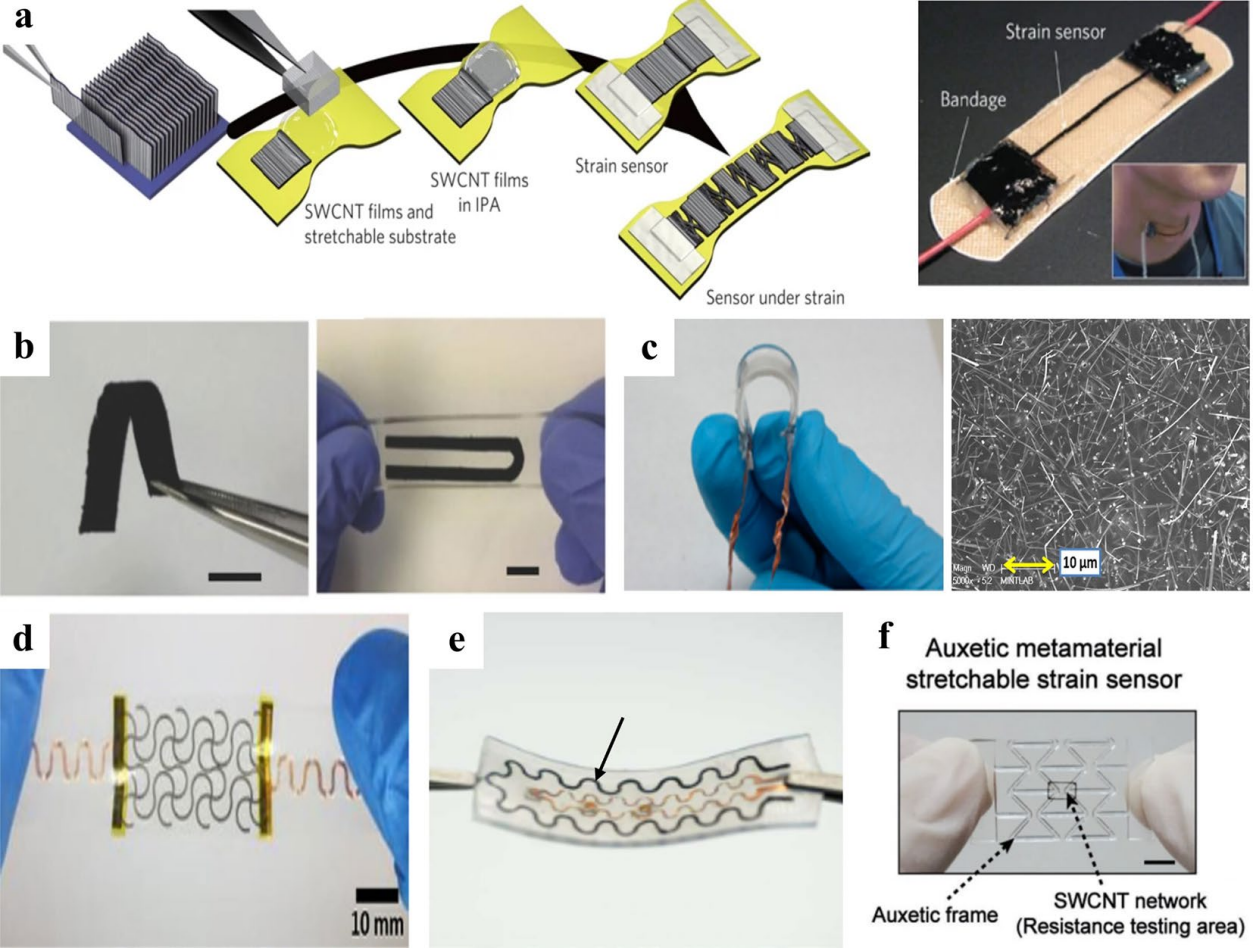
Typical design strategies of flexible strain sensors. **a** Thin films of aligned single-walled carbon nanotubes (SWCNT) [54]. Copyright (2011) Springer Nature. **b** Thin films of graphene-nanocellulose composites [122]. Copyright (2013) Wiley–VCH. **c** Transparent films of sandwich-structured PDMS/AgNW/PDMS nanocomposites [123]. Copyright (2014) American Chemical Society. **d** A serpentine layout of PVDF [124]. Copyright (2019) American Chemical Society. **e** A serpentine layout of hollow Ag-microspheres/carbon nanoparticles/Eco-flex composites [37]. Copyright (2022) Elsevier. **f** Auxetic metamaterial structures regulated SWCNT network on PDMS thin film [125]. Copyright (2018) Wiley–VCH

A flexible strain sensor is used to measure the deformation of objects. Flexible strain sensors play an important role in monitoring human body motions in different positions (Fig. 3b), which can be divided into two categories: (1) motions with large skin deformation, including bending movements of fingers, wrists, arms, legs, and spinal; (2) motions with small skin deformation, including subtle movements of the face, chest, and neck which are directly related to emotional expression, breathing, speaking/swallowing activities, respectively. The latter kind of sensor to detect small deformation is sometimes also referred to as flexible pressure sensors as it is hard to distinguish whether the pressure or the strain stimulus dominates in giving rise to the electrical signal change of the sensors. Flexible strain sensors are usually attached directly to human skins or on clothes and they enable wide applications, such as recording hand gestures [23, 54, 112–114], capturing body movements [115–117], analyzing facial expressions [37, 94, 118, 119], diagnosing throat diseases [95, 120], and monitoring skin sclerosis [121]. Typical design strategies of flexible strain sensors include developing thin films (Fig. 5a–c) and 2D patterns (Fig. 5d–f).

### Flexible Vibration Sensors

**Fig. 6:**
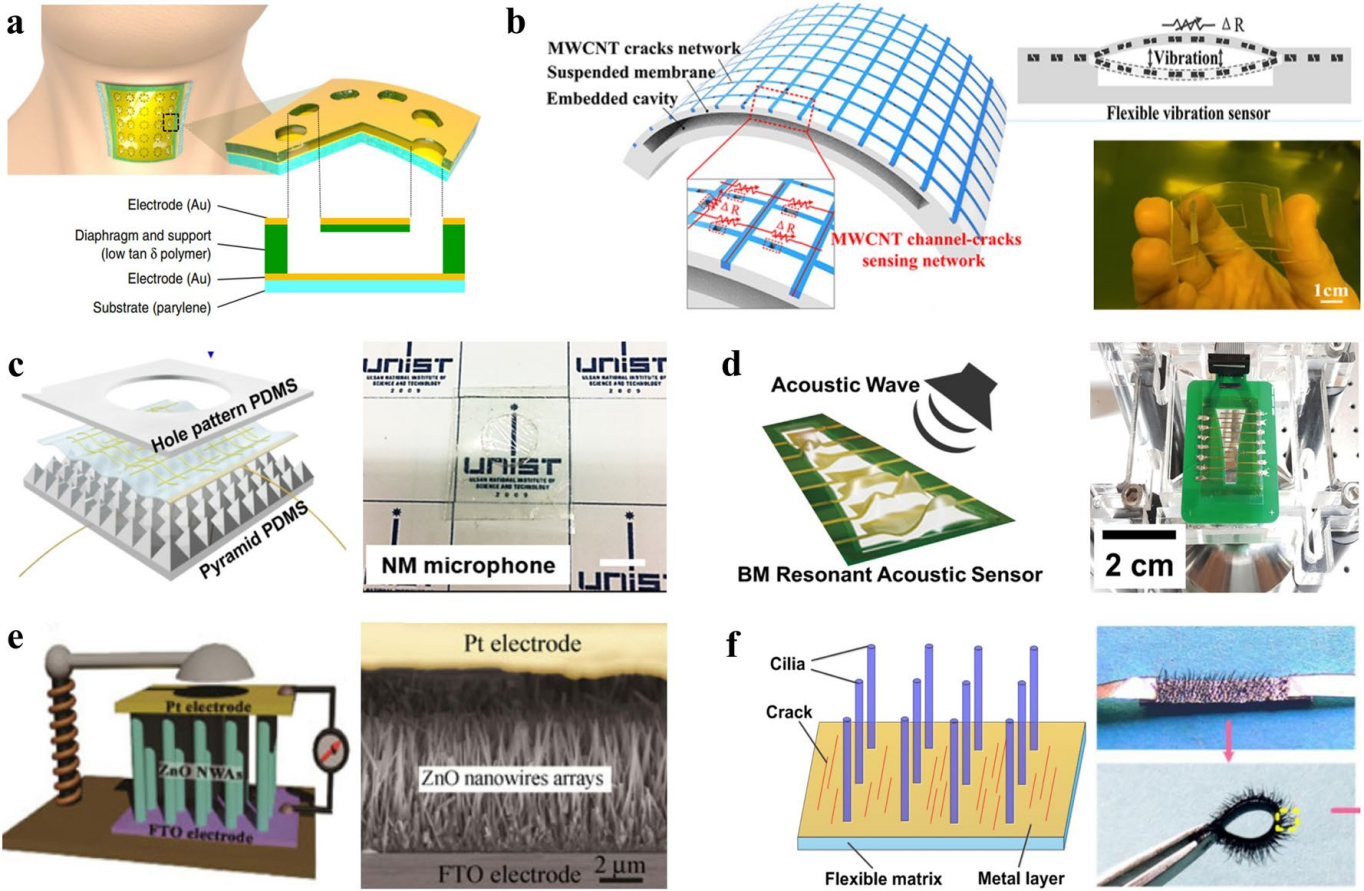
Typical design strategies of flexible vibration sensors. **a** An ultrathin polymer film and a hole-patterned diaphragm structure [97]. Copyright (2019) The Authors. **b** A channel-crack-sensing membrane on a cavity substrate [129]. Copyright (2021) American Chemical Society. **c** A freestanding hybrid nanomembrane on a holey PDMS film and micro pyramid-patterned PDMS film [140]. Copyright (2018) The Authors. **d** A thin PZT membrane on a printed circuit board with a curved shape hole [136]. Copyright (2018) Elsevier. **e** ZnO nanowire arrays sandwiched by two electrodes [133]. Copyright (2013) Springer Nature. **f** Cilium arrays on cracked metal layers [134]. Copyright (2020) American Chemical Society

Flexible sensors that are sensitive to vibrations are of great importance for detecting dynamic mechanical stimuli in various real-world applications (Fig. 3c) such as structural health monitoring [126–129], environmental monitoring [45, 99, 130], acoustic signals recording [18, 19, 27, 131], and medical use [96, 132, 133]. Among different sources of vibration, the natural physiological activity of humans induces mechanical waves propagating through the tissues of the body. And capturing corresponding signals with different amplitudes and frequencies hence reveals important information for disease diagnosis and healthcare applications [96]. The vibration signals can be detected via direct impact or acoustics, such as those measured by a stethoscope. Flexible vibration sensors exhibiting a sensitive response over a low-frequency range (0–100 Hz) have been applied in monitoring body orientation (about 0–0.1 Hz), respiration (about 0.1–0.5 Hz), pulse (about 0.4–2 Hz), and changes in body motion (about 0.5–2 Hz) [134, 135]. Flexible vibration sensors for collecting and recognizing human voices are required to work well in the fundamental voice frequency range (80–255 Hz) and the standard telephony bandwidth (300–3400 Hz) [19, 97, 136]. Although these sensors for low-frequency sensing are also often referred to as flexible pressure sensors, vibrations features including frequency, amplitude, and acceleration are paid more attention to when using the term ‘vibration sensors’. Flexible vibration sensors with an ultra-high operating frequency range from several kHz to several MHz are highly desirable for ultrasonic-based structural health monitoring [98, 137–139]. Typical design strategies of flexible vibration sensors include designing suspended membranes (Fig. 6a–d) and vertical arrays of micro/nanowires (Fig. 6e, f).

### Flexible Shear Sensors

**Fig. 7:**
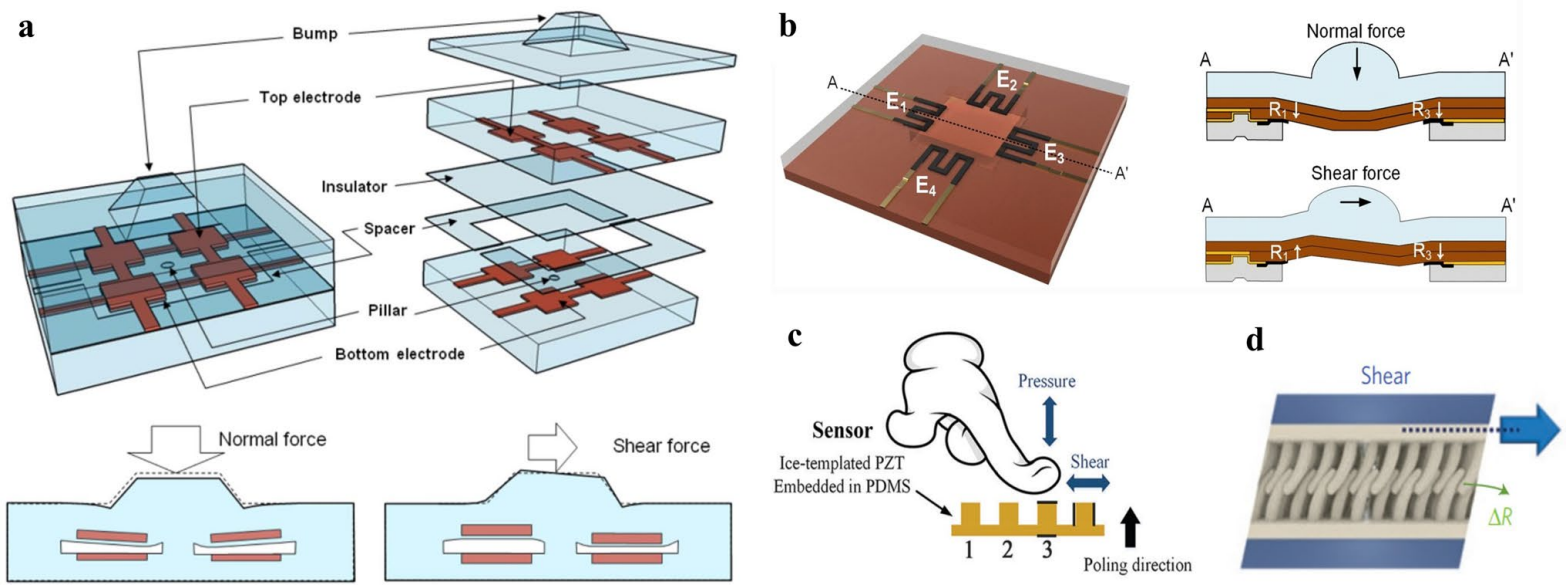
Typical design strategies of flexible shear sensors. **a** A bump with four distributed underlying capacitive sensing elements [148]. Copyright (2008) IEEE.** b** A bump with four distributed underlying resistive sensing elements [152]. Copyright (2019) The Authors. **c** A surface pillar vertically sandwiched by two electrodes [146]. Copyright (2018) The Authors. **d** Interlocking arrays of nanofibers [153]. Copyright (2012) Springer Nature

Flexible sensors to perceive shear stress (Fig. 3d) play an important role in monitoring fluidic dynamics [141–143], the biomedical field [144, 145], and robotics [146–149]. Real-time shear-stress information is critical in estimating the airflow situation on the surface of aircraft to adjust the flight control correspondingly. For example, a 1D array of flexible shear sensors has been developed to detect the leading-edge flow separation point of unmanned aerial vehicles to guide the independent flight control of pitching, rolling, and yawing via force imbalance [141–143]. Similarly, shear-stress monitoring in blood vessels promotes the understanding of the relationship between blood flow and vascular disease [145]. The increasing demand for measuring shear stress in the medical community can also be found in the significance of analyzing interfacial forces between the human body and external objects, such as measuring the friction by flexible sensors between a prosthesis and a stump to check its fitness [100, 144]. Moreover, tactile sensors with the capability of sensing shear stress can provide robotics with direct information on textures or slip detection [146–150]. Flexible shear sensors have been realized by various sensing mechanisms including piezoresistive [151–153], piezocapacitive [148, 154], piezoelectric [146, 155], triboelectric [156, 157], magnetic [158], ferroelectric [159], or optic shear sensors [144, 160]. Typical design strategies of flexible shear sensors include using a bump on the sensor’s surface with four distributed underlying sensing elements (Fig. 7a, b) and deformable surface/internal microstructures (Fig. 7c, d) to perceive shear stimuli.

### Flexible Multimodal Sensors

Flexible sensors that are capable of perceiving and decoupling different types of stimuli including forces, temperature, humidity, etc., are currently one of the research focuses for a wide range of applications including robotics, health monitoring, and HMI [161–166]. Compared with sensors for a single stimulus, flexible and multimodal mechanical sensing platforms have a distinct advantage of capturing comprehensive information of pressure, strain, vibration, shear, and other mechanical stimuli to realize complicated tasks, whereas cross-sensitivity must be diminished for accurate measurements. For example, tactile sensors capable of sensing normal pressure and shear stress at the same time are essential parts of e-skins to provide robotics with information for complex object recognitions and dexterous object manipulation [146–150]. Constructing the array layout of identical sensing units, integrating different sensing units, and developing novel materials with multiple sensing mechanisms are all effective approaches to decouple different mechanical signals [167].

## ML-Assisted Data Interpretation

**Fig. 8:**
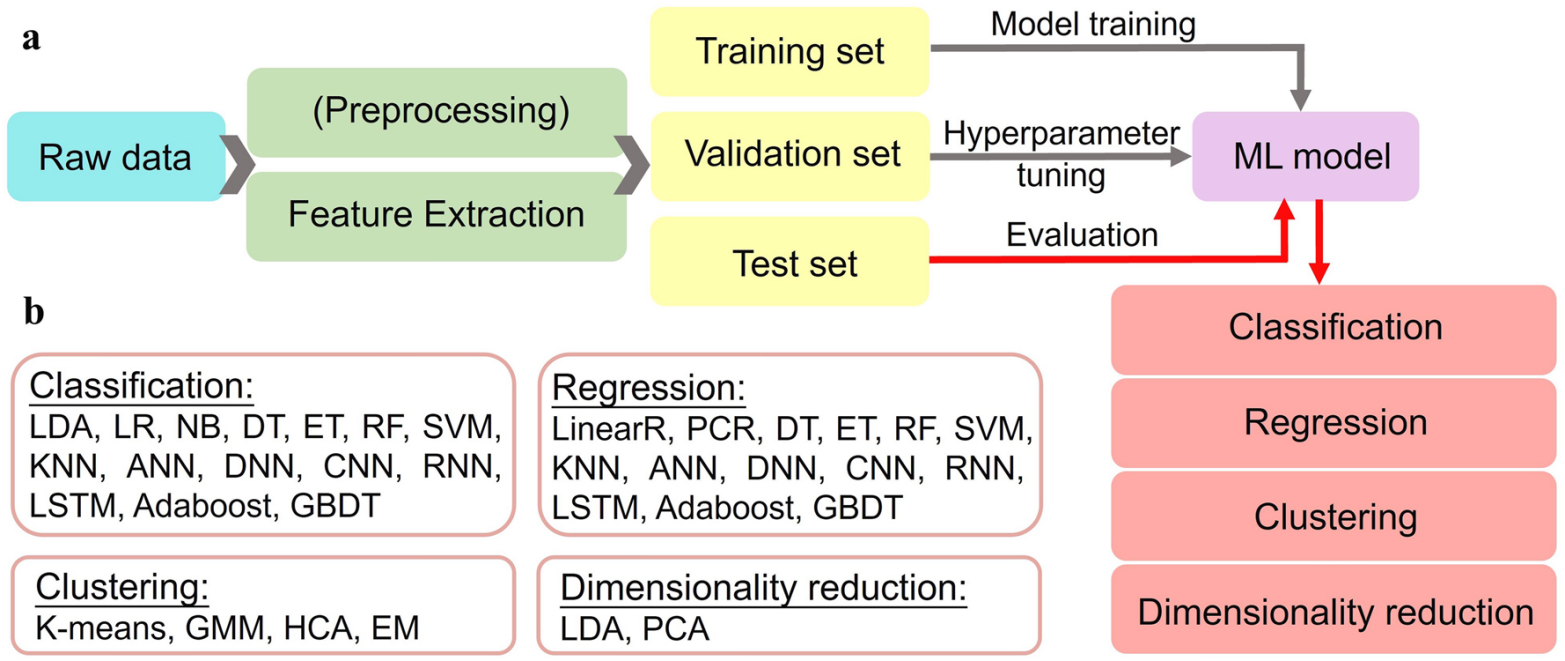
The general process of ML techniques for data interpretation. (b) Common ML algorithms for classification, regression, clustering, and dimensionality reduction. LDA: Linear Discriminant Analysis. LR: Logistic Regression. NB: Naive Bayes. DT: Decision Tree. ET: Extra Tree (Extremely Randomized Tree). RF: Random Forest. SVM: Support Vector Machine. kNN: k-Nearest Neighbor. ANN: Artificial Neural Network. DNN: Deep Neural Network. CNN: Convolutional Neural Network. RNN: Recurrent Neural Network. LSTM: Long Short-Term Memory. Adaboost: Adaptive Boosting. GBDT: Gradient Boosting. LinearR: Linear Regression. PCR and PCA: Principal Component Regression/Analysis. GMM: Gaussian Mixture Model. HCA: Hierarchical Clustering Analysis. EM: Expectation Maximization

ML is defined as a computer program that possesses the ability to acquire knowledge by extracting features from raw data and using the gained knowledge to answer real-world problems. When ML is introduced to flexible sensing technology, it profoundly impacted this field by adding a strong tool for processing and analyzing data from a single sensor or multiple/multimodal sensors. Supervised learning and unsupervised learning are the most commonly used ML algorithms in dealing with flexible mechanical sensing data. For supervised learning, a group of input data with their given outputs is utilized to train ML algorithms, which can perform classification or regression tasks (predict a discrete class label or continuous quantity). By comparison, unsupervised learning only has unlabeled training datasets and is always used to cluster datasets into a group. The common ML algorithms for these purposes have been shown in Fig. 8.

SVM, tree-based algorithms, and neural network-based algorithms are the three mostly used ML algorithms. SVM is widely favored in classification problems as it produces notable correctness with great simplicity. It is very productive in dealing with high-dimensional spaces and requires small sample size. SVM is effective for both nonlinear and linear discrimination. However, SVM is not suitable for large data sets. And it also does not execute well when dataset is incomplete or noisy. DT works well in both classification and regression problems, and it is very intuitive and can be easily understood. DT needs less efforts for data preprocessing since neither data normalization nor missing value treatment is required. One of the most common disadvantages of DT is that it can easily overfit. DT algorithm also has the disadvantage of instability since it is sensitive to small changes in the data. This makes it highly susceptible to data drifts and unsuitable to be used over long periods. This limitation can be solved by other tree-based algorithms such as RF, but they lose interpretability.

ANN is the most popular ML algorithm since it has enhanced learning ability and adaptive nature. It is capable of comprehensively learning very complicated relationships. Besides, ANN has strong robustness to noise and fault tolerance. But it takes huge sets of data and lots of time to train an ANN. How the results are achieved is difficult to understand. It is also difficult to determine the proper network structures with many parameters, which are decided through experience and trial and error. DNN is an advanced ANN (also known as shallow neural network) to possess multiple hidden layers between the input and output, which allow it to extract richer data features from more complicated relationships and ultimately improve the accuracy of classification/prediction. Compared with traditional ANN, DNN requires larger amounts of training data and much higher computing power. CNN and RNN are two classes of DNN. CNN is effective in automatically capturing spatial features whereas RNN has been developed to capture time-series information from the input data. LSTM is a modified version of RNN, which makes it easier to learn long-term dependencies.

There is a general process of ML techniques used for data interpretation of flexible mechanical sensors. Based on the recorded sensor signals and the ultimate goal of intelligent sensing systems, an appropriate ML model should be designed above all. Once the ML model is initially built up, the collected raw data commonly require preprocessing before they are transformed into features that can be used for ML analyses. The general preprocessing methods contain removing outliers, denoising, transformations, normalizations, and so forth. This step is critical since improper preprocessing may also lead to the loss of some informative features of raw data. Then the obtained dataset should be divided into three subsets, namely the training set (usually 60%), validation set (usually 20%), and test set (usually 20%) [168]. The training set of data is used to determine the parameters of the selected ML model. Validation data set is applied to evaluate the model fit on the training set and accordingly tune the hyperparameters of the model. In some cases, the validation set is ignored and thus training set occupies about 80% of the total dataset. After the final ML model is determined by the training and validation set, its performance is reported by the test data set. Take the ML model for classifications as an example, the accuracy evaluated by the test data set can be displayed in a confusion matrix. The following sections are the integration of flexible mechanical sensing technology with ML-assisted data interpretation for various applications, out of which some representative works are listed in Table 2.

**Table 2:** Representative works of ML-assisted data interpretation in flexible mechanical sensing technology (NA: not available)

Field	Applications	Data source	Sensor type	ML algorithms	Accuracy
Health monitoring	Blood pressure estimation	Pulse signal	A triboelectric sensor [14]	ANN	NA
	Pulse pattern classification	Pulse signal	A piezoelectric sensor [13]	DTW	NA
	Respiration pattern classification	Respiration signal	A triboelectric sensor array [169]	CNN	NA
	Swallow volume/swallow function identification	Submental motion and electrical activity of the swallowing muscles	A piezoresistive strain sensor and sEMG electrodes [16, 17]	L1-distance	92%, 6 volumes
	Mental fatigue levels identification	ECG signal, respiration, galvanic skin responses	A piezoresistive strain sensor, surface electrodes [170]	DT	89%, 3 fatigue levels
HMI of voice communication	Speaker recognition	Acoustic signal	Multi-channel piezoelectric sensor [18, 19]	GMM + EM	97.5%, 40 speakers [14]
	Speaker/voice commands recognition	Acoustic signal	A triboelectric sensor [20]	DTW	98%, 5 words
	Silent speech recognition	Face deformation	Two piezoresistive sensors [172]	CNN	88%, 100 words
	Silent speech recognition	Throat movements	A piezoresistive sensor [173]	CNN	70%, 3 words
	Silent speech recognition	Electrical activity of face	sEMG electrodes [174]	LDA	93%, 110 words
HMI of hand gesture identification	Sign language recognition	Finger motion	5 triboelectric strain sensors [23]	SVM	99%, 660 gestures
	Gesture recognition in VR/AR	Finger motion	5 triboelectric pressure/strain sensors [175]	CNN	99%, 3 gestures
	Sign language recognition	Finger motion	15 piezoresistive strain sensors [176]	ANN	99%, 8 gestures
	Hand motion recognition	Wrist skin deformation	A single piezoresistive strain sensor [177]	DNN	96%, 8 motions
	Sign language recognition of 26 letters	Wrist muscle movement	A triboelectric and piezoelectric sensor array [178]	LDA	93%, 26 gestures
	Gesture recognition	Finger motion	5 piezoresistive strain sensors (integrated with visual data) [114]	ANN	100%, 10 gestures
	Hand gesture recognition	Electrical activity of the forearm muscles	sEMG electrodes [24]	HD computing	93%, 21 gestures
Object/surface recognition	Gripped object recognition	Tactile pressure	548 piezoresistive sensors [34]	CNN	NA, 26 objects
	Gripped object recognition	Tactile pressure and finger motion	A tactile TENG sensor array and a length TENG sensor [179]	SVM	98%, 16 objects
	Gripped object recognition	Tactile pressure, material thermal conductivity, object/environment temperatures	A quadruple tactile sensor array [180]	ANN	94%, 7 objects
	Texture classification	Rubbing textures with roughness	A piezoresistive pressure sensor array and a TENG pressure sensor [181]	ANN	99%, 12 fabrics
	Texture classification	Rubbing textures with roughness	A piezoresistive and a piezoelectric pressure sensor [182]	ANN	99%, 20 fabrics
	Texture classification	Touching textures with roughness	A piezoresistive pressure sensor array [22]	CNN	94%, 10 textures
Pressure prediction and position recognition	Pressure differentiation	Applied pressure	3 LC pressure sensors [183]	CNN	NA
	Pressure distribution estimation	Applied pressure	A piezoresistive sheet [185, 186]	DNN	NA
	Pressure resolution improvement	Applied pressure	A magnetic sensor array [158]	ANN	NA
	Impact position estimation	Impact force	A piezoelectric sensor array [99]	CNN	NA
Human posture/motion identification	Human post/motion classification	Tactile pressure	Piezoresistive pressure sensor arrays [187]	CNN	100%, 10 lying postures/supporting surfaces
	Activity classification	Body temperature	A temperature sensor array [188]	CNN	96%, 4 activities
	Sitting posture classification	Sitting pressure	8 × 8 piezoresistive pressure sensors [189]	RF and ANN	97%, 6 postures
	Sitting posture classification	Sitting pressure	10 triboelectric pressure sensors [1]	CNN	97%, 6 users
	Individual recognition by smart mat	Walking pressure	3 × 4 triboelectric pressure sensor arrays [21]	CNN	96%, 10 users

### Health Monitoring

**Fig. 9:**
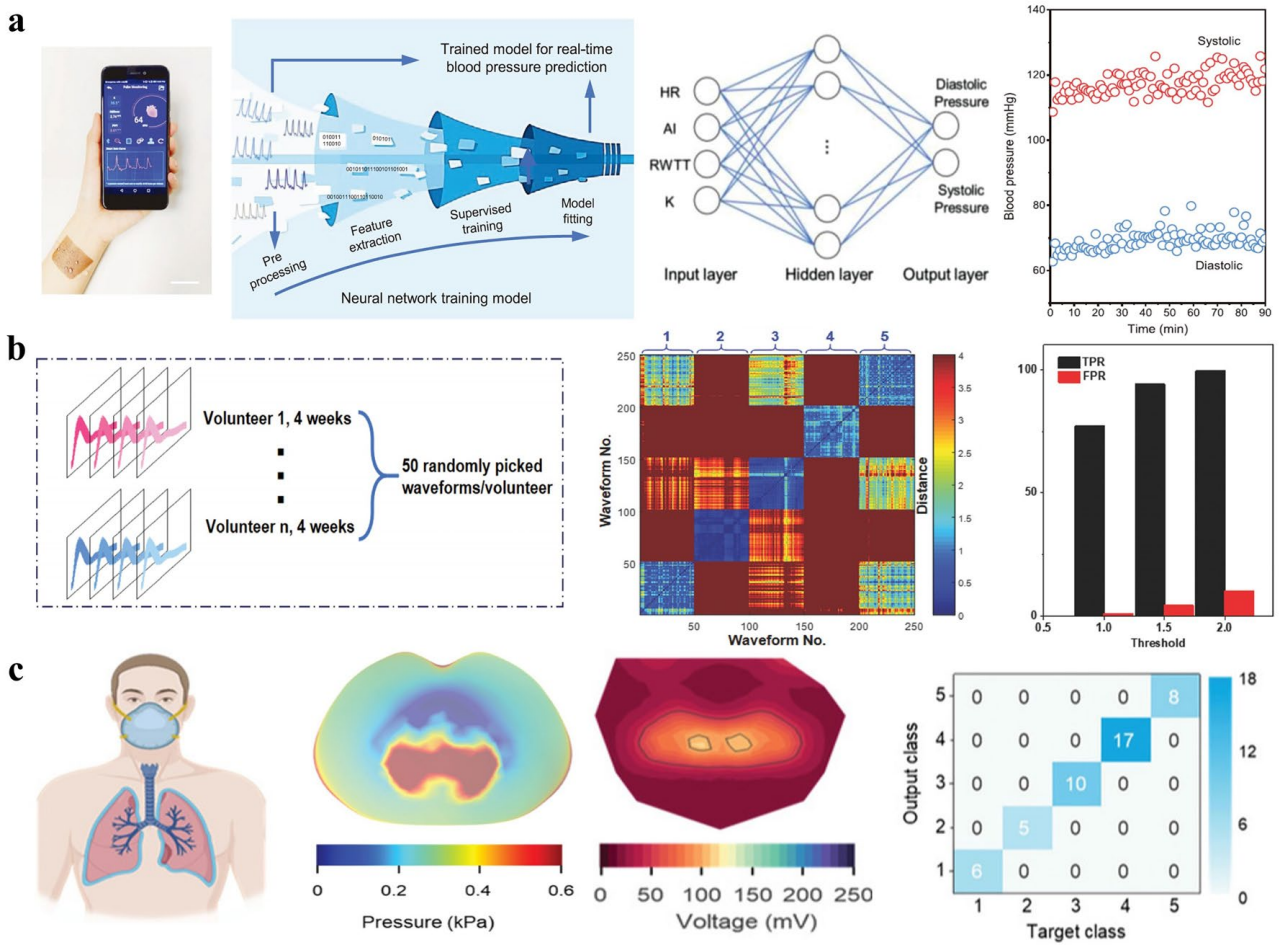
**a** Blood pressure estimation using a textile triboelectric sensor assisted by ML techniques [14]. Copyright (2021) Wiley–VCH. **b** Pulse wave differentiation using a wearable piezoelectric sensing system assisted by ML techniques [13]. Copyright (2021) Wiley–VCH. **c** Respiration pattern differentiation using an on-mask triboelectric sensor network assisted by ML techniques [169]. Copyright (2022) Wiley–VCH

Flexible mechanical sensing systems that couple intimately to the human skin is becoming a popular tool for convenient, real-time, and continuous detection of numerous physiological signals, which have been widely analyzed by ML algorithms for further understanding of our health status. ML-assisted signal analyses in human pulse monitoring by wearable pressure sensing systems is a typical example. Reportedly, Chen et al. developed a textile triboelectric sensor for monitoring arterial pulsatility and used the ML technique to predict blood pressure from the recorded pulse signals, as shown in Fig. 9a [14]. Pulse wave features were extracted as inputs for a supervised feedforward neural network, which generates two outputs, namely systolic and diastolic blood pressure. The estimated values present small mean deviations of 2.9% and 1.2%, respectively, from the values measured by commercial cuffs. Similarly, Yang et al. employed three different ML algorithms, namely RF regression, GBTD regression, and Adaboost regression, to estimate systolic and diastolic blood pressure from the measured pulse-wave signals of the proposed devices [15]. Among these algorithms, the RF regression-based algorithm proved the best performance. Moreover, Lin et al. adopted ML technology based on a dynamic time warping (DTW) algorithm for the classification of pulse wave patterns by a wearable piezoelectric pulse sensing system (Fig. 9b), which is critical in pulse diagnosis since pulse waves usually remain periodically steady for each person and obvious change implies underlying health issues [13]. The classification results show high similarity between the pulse waves of the same volunteer, indicating stability in their pulse features for the test period. In contrast, the highest dissimilarity was demonstrated between different volunteers’ pulse waves, proving the excellent precision and stability of the pulse sensing system to collect health data from different users in the long term. Similar to pulse monitoring, Chen et al. developed an on-mask respiratory monitoring system by textile triboelectric sensors and used CNN-based ML technique (Fig. 9c) to recognize different respiration patterns for real-time respiratory diagnosis [169].

Synergistically, when integrated with ML methods, simultaneous measurements of different sensors can be combined to automatically proffer useful health information. Lipomi et al. integrated the strain sensor and surface electromyography (sEMG) electrodes on the neck to provide data for the ML algorithms based on L1 distance in identifying different swallowed objects/volumes [16, 17]. These works hold great promise for various applications such as swallowing function monitoring, nutrition science, and sports medicine. Zang et al. designed a multimodal epidermal electronic system placed on the chest and a palm for simultaneous detection of electrocardiogram and respiration rate, as well as galvanic skin response, with the obtained signals analyzed by the DT algorithm to determine the mental fatigue levels [170]. Moreover, Jeong, Rogers, and Xu proposed a continuous on-body sensing system to detect COVID-19-related symptoms and linked the data with other testing results for the development of ML techniques to assess COVID-19 infection and recovery [35]. By contrast, ML can also help recognize different stimuli from the signals of a single multifunctional sensor. Sahatiya et al. adopted ET to decouple strain, pressure, and breath stimuli from the collected data of the developed SnS_2_ QD/PVA sensor, which is multifunctional and water soluble to offer promising opportunities in flexible and eco-friendly transient medical electronics [171]. Especially, Lu et al. considered the patients’ privacy when using the ML to classify health conditions based on the electrocardiogram (ECG) data in the body sensor network, and thus exerted selective encryption schemes to protect them against illegal classification on the attacker side [190].

### HMI of Voice Communication

ML algorithms have also been brought into HMI of voice communication by integrating with various flexible acoustic pressure sensors to strengthen their functionalities. To acquire the entire human speech frequency range, Lee et al. proposed a seven-channel flexible piezoelectric acoustic sensor for speaker identification by ML algorithms based on a GMM followed by EM, and the multi-channel sound inputs were demonstrated to provide abundant voice information [18]. Thereafter, the same group further broadened the resonant bandwidth of the piezoelectric acoustic sensors by adopting a biomimetic frequency band control method, which also improved the sensitivity in a miniaturized dimension for accurate biometric authentication via the same ML algorithms [19]. In addition to speaker recognition, Chen et al. designed a flexible acoustic sensor based on microparticle vibrations and surface triboelectrification, thereby enabling the recognition of not only speakers but also simple voice commands as the collected data was processed by the DTW algorithm [20]. Han et al. fabricated flexible piezoresistive pressure sensors and integrated them with the single-layer perceptron model algorithms to perform music recognition [131].

**Fig. 10:**
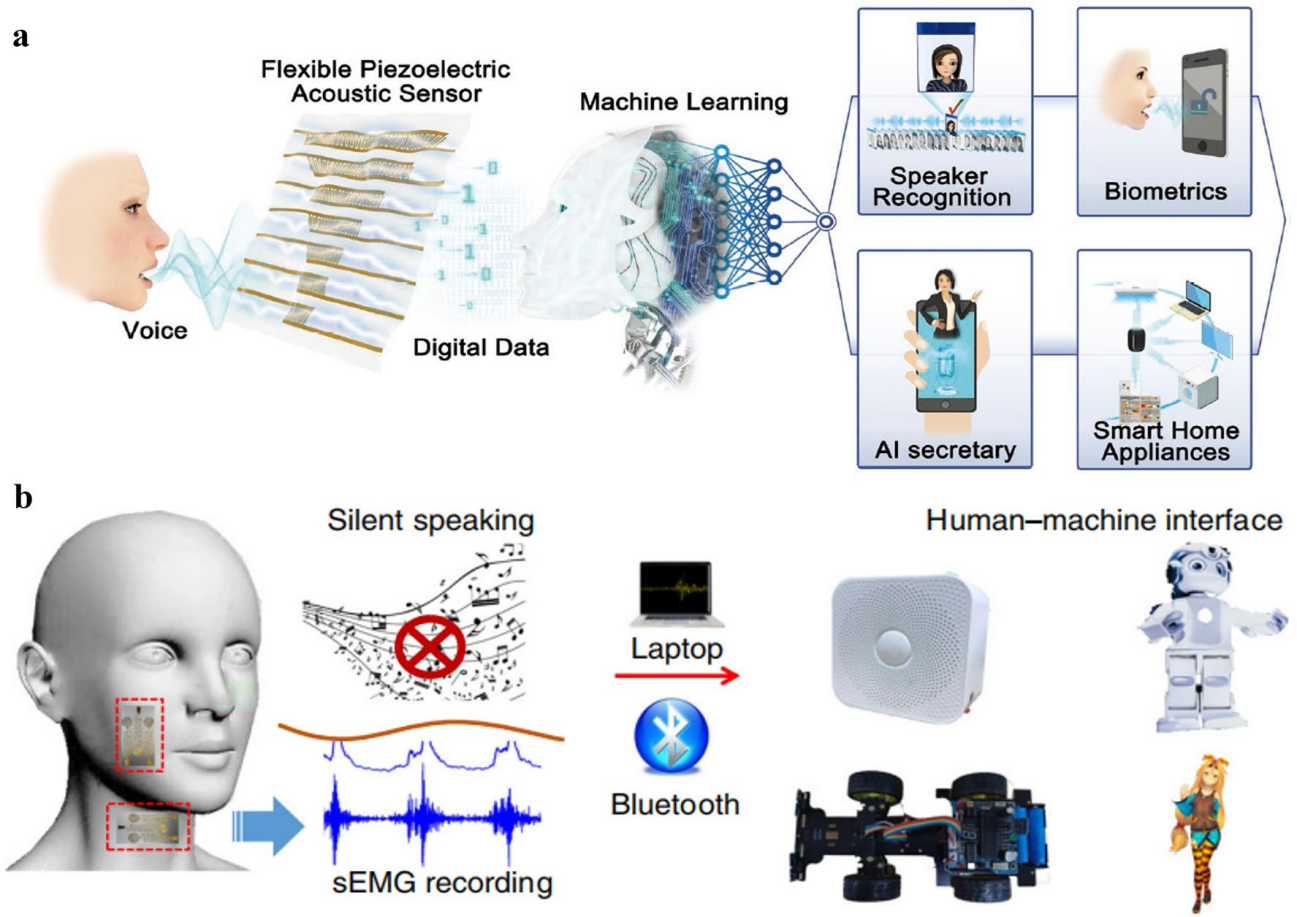
**a** HMI applications of voice communication enabled by the flexible piezoelectric pressure sensor and ML techniques [27]. Copyright (2019) Wiley–VCH. **b** HMI applications of silent speaking enabled by the flexible sEMG sensors and ML techniques [191]. Copyright (2020) The Authors

Moreover, ML has also assisted intelligent silent recognition when combined with flexible sensors on the skin at locations of faces and necks, which have great potential in helping patients that are losing their voice. Lin et al. developed a pressure sensor of resistive type to detect the throat movements of saying different instructions without the real sounds coming out, and CNN was adopted to recognize the recorded signals [173]. Recently, Yu et al. developed a silent speech interface by using crystalline silicon-based strain sensors on the face, and combined a CNN algorithm to realize the recognition of 100 words at a high accuracy rate (87.53%) [172]. Lee et al. proposed unique sEMG sensors on the jaw and face to collect from three muscle channels and finally realized silent speech recognition of simple instructions (Fig. 10b) by LDA algorithms [191]. Similarly, Huang et al. adopted four-channel sEMG sensors on the face and LDA algorithms but recognized up to 110 words covering daily vocabularies [174].

### HMI of Hand Gesture Identification

**Fig. 11:**
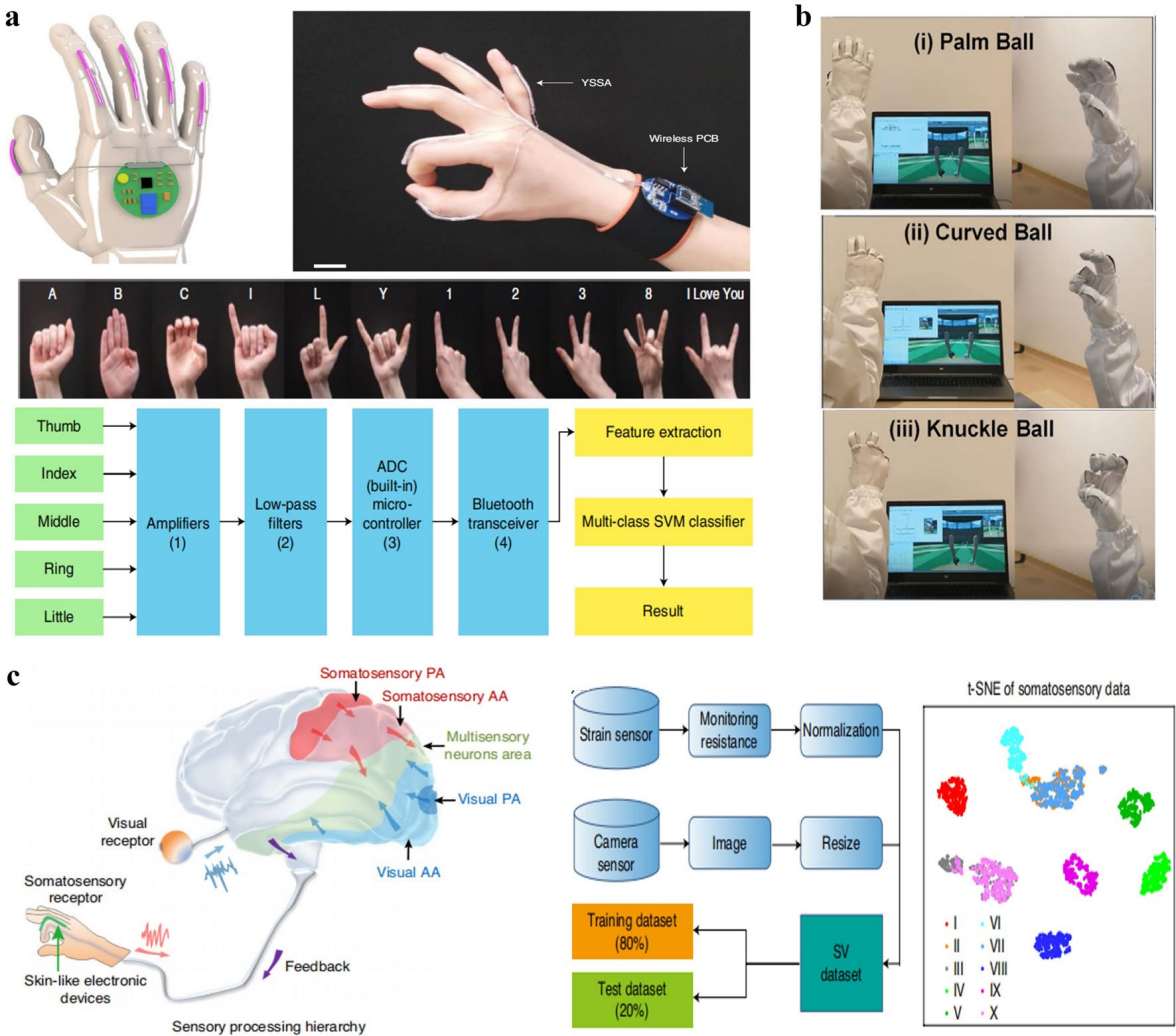
**a** HMI applications of sign language translation enabled by a stretchable strain sensor array on fingers and ML techniques [23]. Copyright (2020) Springer Nature. **b** HMI applications of VR control enabled by smart gloves and ML techniques [175]. Copyright (2020) The Authors. **c** HMI applications of hand gesture recognition enabled by analyzing visual data and somatosensory data from stretchable strain sensors on fingers via ML techniques [114]. Copyright (2020) Springer Nature

ML-assisted data analyses have also facilitated HMI applications of different sensing arrays attached on/near human hands or in the forms of smart gloves to recognize hand gestures for sign language translation, understanding grasp action, VR/AR control, etc. As shown in Fig. 11a, Chen et al. proposed a simple design of five stretchable strain sensors of triboelectric mechanism attached to human fingers to monitor each finger motion and utilized SVM to translate the detected signals of hand gestures of American Sign Language into speech [23]. Similarly, Lee et al. combined five superhydrophobic triboelectric textile sensors on fingers and CNN to realize the recognition of several gestures in VR/AR applications (Fig. 11b) [175]. Especially, Li et al. designed six resistive strain sensor units covering the main tendons of the hand back to sense their deformation, which provided information for the SVM method to recognize twelve typical precision-grasping gestures [192]. To get more detailed information on hand motion in each part, more flexible sensors have been demonstrated. Park et al. fabricated 15 stretchable resistive strain sensors on finger joint regions and utilized ANN for translating hand sign language [176]. Thean et al. developed 16 bimodal capacitance sensors distributed close to the joints of the human palm and integrated a LSTM network to achieve both static and dynamic hand gesture recognition [193].

Different from the above on-hand sensor arrays, Ko et al. developed a single resistive skin sensor detecting minute skin deformations of the wrist and analyzed its signals by DNN algorithms to determine dynamic hand motions [177]. The sensor is also reported to be capable of extracting gait motions when attached on pelvis. Wang et al. proposed a wristband-style mechanical device based on a hybrid sensor array with 8 triboelectric and piezoelectric sensors to capture mechanical information regarding hand movement and adopted LDA algorithms for gesture recognition [178]. Rabaey et al. realized gesture recognition by monitoring electrical muscle activities of the forearm, of which the entire circumference was wrapped by a large-area, high-density sEMG electrode array with 64 channels, and a neuro-inspired hyperdimensional (HD) computing algorithm was adopted to realize in-sensor adaptive learning and real-time inference [24]. Further, beyond merely taking advantage of flexible sensing technology, multimodal fusion was proposed by Chen et al. by integrating visual data with somatosensory data from five stretchable resistive strain sensors on fingers to classify hand gestures (Fig. 11c) [114]. A sparse neural network was used for data fusion and recognition at the feature level, thus achieving a recognition accuracy as high as 100%.

### Object/Surface Recognition

**Fig. 12:**
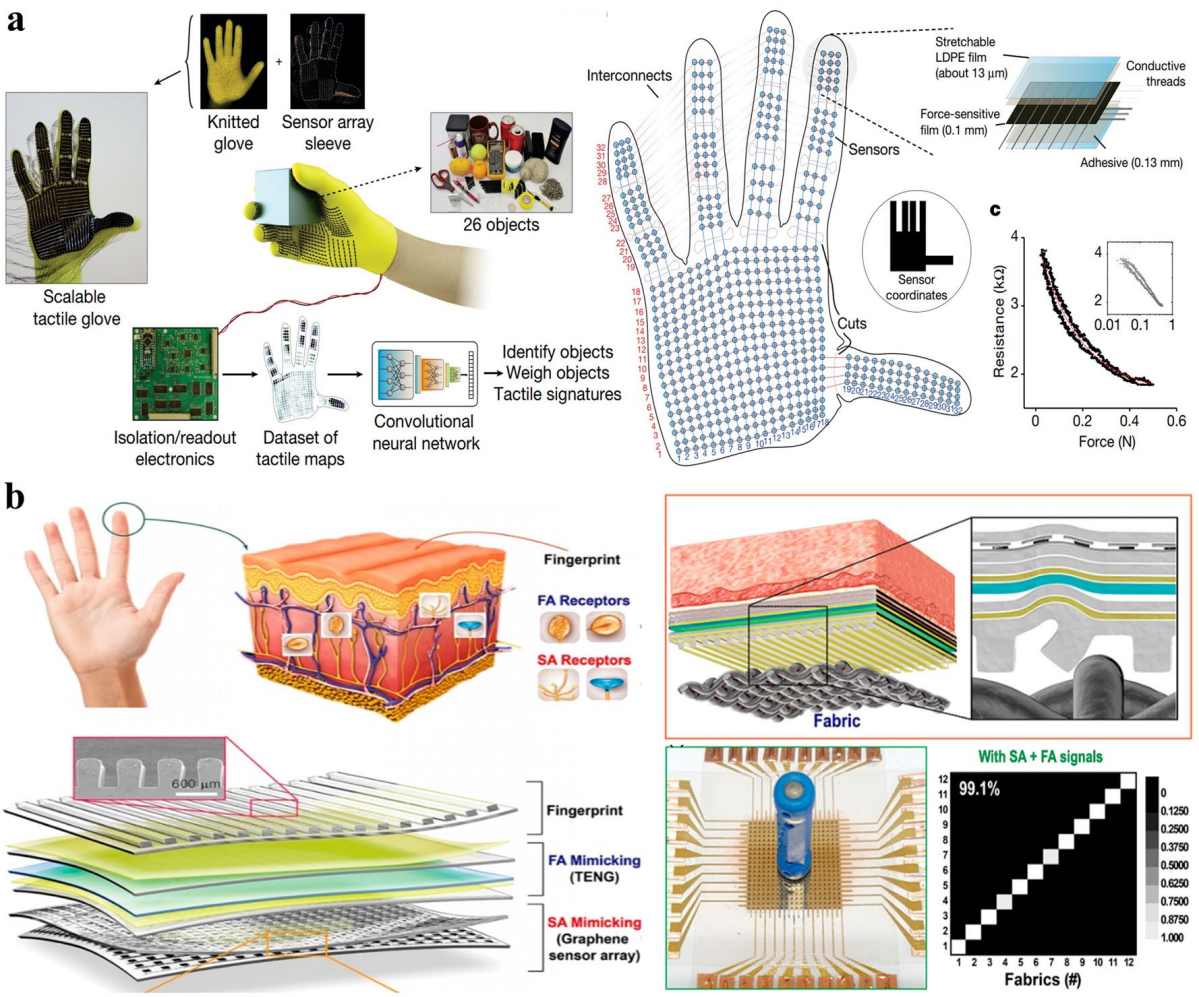
**a** Object recognition and weighing enabled by a smart glove and ML techniques [34]. Copyright (2019) Springer Nature. **b** Texture recognition realized by SA- and FA-mimicking sensors and ML techniques [181]. Copyright (2019) American Chemical Society

The acquirement of tactile maps on hands has also been processed by the ML method to identify objects and infer their properties. Matusik et al. developed a tactile glove with uniformly distributed 548 piezoresistive sensors, of which the array data was analyzed by CNN (Fig. 12a) to identify individual objects and estimate their weight [34]. It should be noted that a linear model was compared with the CNN in predicting weight, proving that the latter performs better over the entire weight range. The CNN presents an average prediction error of 56.88 g whereas the linear algorithm possesses 89.68 g. The relationship between the object weight and tactile signals is complex and it is significant to introduce ML algorithms. Besides, ML is also effective for dealing with signals of hybrid sensor systems. Lee et al. proposed a smart soft gripper integrated with a tactile TENG sensor array and a length TENG sensor for each finger, and the collected data was processed by an SVM-based analytic platform for gripped object recognition [179]. Similarly, Rus et al. also fabricated a dual-modality sensing glove, which consists of 16 resistive strain sensors and 6 resistive pressure sensors, to monitor data for both hand pose reconstruction and object identification by ANN algorithms [194]. Further, Zhu et al. developed quadruple tactile sensors on a robotic hand to perceive thermal conductivity, contact pressure, as well as object and environment temperature simultaneously and independently [180]. The multimodal sensing information was fused by a feedforward ANN to achieve precise recognition of object size, shape, and material.

Different from the above flexible tactile sensing arrays distributed over hand, artificial fingertip tactile sensors have been assisted by ML to distinguish different materials merely based on their surface textures. Most early tactile sensors are embedded into a flexible artificial finger, which is sometimes even designed with biomimetic fingerprints on the surface to enhance tactile sensitivity in some works. Oddo et al. developed an array of four microelectromechanical systems (MEMS) tactile microsensors in polymeric packaging, which is similar to human Merkel mechanoreceptors of the human finger, with a kNN classifier to differentiate simple textiles with periodic texture [195]. Sammut et al. utilized randomly distributed strain gauges and piezoelectric sensors embedded in silicone to provide information for several ML algorithms for comparison to identify widely different materials and the same material of different textures [196]. The result shows that boosting on naive Bayes tree achieves the best performance among others. Loeb et al. fabricated a multimodal tactile sensor with a piezoresistive pressure sensor to measure tactile vibration and impedance sensing electrodes to measure force, and Bayesian exploration was used for textural property identification including traction, roughness, and fineness [197]. Peters et al. proposed a bioinspired artificial fingertip consisting of two piezoelectric sensors for acquiring tactile signals and used SVM to discriminate material texture based on different surface roughness [198].

Recently, intrinsically flexible film tactile sensors based on flexible mechanical sensing technology have been developed to mimic the tactile functions of human skins for surface texture identification and thus can be applied on the surface of a prosthetic hand. Joen et al. presented a flexible piezoresistive pressure sensor and utilized an LSTM network to recognize patterns of current change of the sensors when rubbing different textures [199]. In an attempt to mimic the slow adaptive (SA) and fast adaptive (FA) mechanoreceptors in finger skin, Chun et al. proposed a tactile sensor consisting of a piezoresistive sensor array and a TENG sensor for pressure- and vibration-sensitivity (Fig. 12b), which were both combined to provide information for ANN algorithms to classify different fabrics possessing complex patterns with aperiodic roughness [181]. It should be noted that the combination of SA and FA sensor signals was proved to improve the classification rate, compared to using a single signal kind. Later, Chun et al. further reported a neural tactile sensing system with artificial SA and FA mechanoreceptors using piezoresistive and piezoelectric particle-based sensors, and ANN algorithms were still adopted to classify different fabrics [182]. Tee et al. [32] also presented a piezoresistive sensor with vibration sensitivity and combined kNN to process its signals for texture recognition. Then, the same group developed a 100-array tactile piezoresistive e-skin as an FA sensor and formed a tactile image by aggregating FA responses of every sensor element to be used for texture classification via CNN [22]. Compared to commonly sliding or exploratory motions for texture detection, the tactile sensors in arrays realized fast and reliable classification of textile surface textures through a one-touch event. Similarly, Luo et al. also proposed a bioinspired tactile sensor array for multipixel sensing, which allows rich information about the environment to be captured based on the triboelectric effect. The feedforward ANN was adopted for the recognition of objects placed on its surface, but instead of using surface texture features, the intrinsic properties of their materials in gaining or losing electrons were utilized as a valuable feature (leading to the difference in the contact electrification effect), as well as the weight and shape of the object [200].

### Pressure Prediction and Position Recognition

**Fig. 13:**
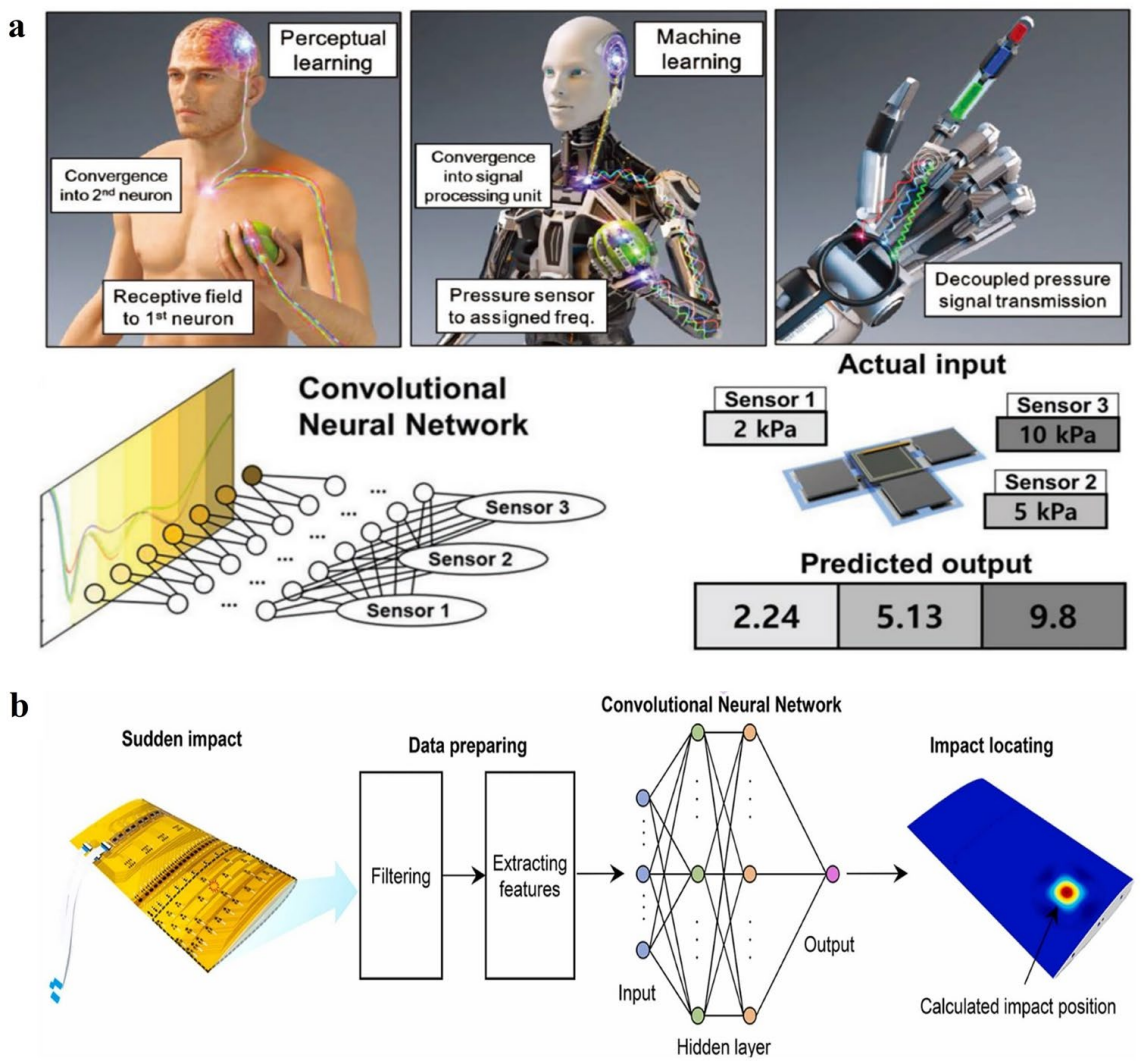
**a** Pressure prediction and position recognition of a pressure-sensing platform, which is inspired by the human somatosensory system and realized by flexible LC pressure sensor arrays and ML-based cognition [183]. Copyright (2019) Wiley–VCH. **b** Impact positioning of the flexible sensing skin for the flying perception of aircraft, realized by a piezoelectric sensor array and ML techniques [99]. Copyright (2021) Elsevier

ML-assisted signal analyses have been applied in the e-skin and other kinds of large-area pressure-sensing devices for pressure prediction and position recognition. On one hand, ML offers an effective way to process the complicated relationship among signals of multiple sensors and remove the crosstalk between them. As shown in Fig. 13a, Park et al. [183] proposed a parallel signal processing scheme for a pressure-sensing system, which was inspired by the human somatosensory system and realized by CNN-based cognition. The pressure signals of the three sensors were uniquely combined into a single output signal pattern, which was similar to that of tactile sensors combined with artificial synaptic devices, and then subsequently processed by CNN to identify the pressure applied to each sensor. Differently, Kim et al. used a flexible tactile sensor array as a Braille reader and predicted the designated letters from the acquired electrical signals based on SVM [184]. It was stated that a translational movement of the Braille letter does not affect the recognition and obviously, there is still room for improvement to work even under rotation.

On the other hand, ML facilitates a simple array-free structure design of large-area pressure sensing platforms while still maintaining accurate pressure distribution estimations or improves the pressure sensing resolution from sensor arrays. Sohn et al. developed bulky macroscale electronic skin by employing a single-layered piezoresistive MWCNT-PDMS composite without array patterns [185]. The resistance changes were measured at the edge of the whole e-skin by multiple probe terminals, of which the data were input into DNN to estimate the pressure value and location. After that, they proposed a similar bulk sheet made of a piezoresistive carbon nanotube (CNT)-Ecoflex composite to be used as a smart keypad [186]. Wang et al. realized non-array tactile sensing technology based on electrical impedance tomography (EIT) and subsequently used a deep learning method to post-process the originally constructed conductivity images to enhance the spatial resolution of sensor tactile perception [201]. Further, Shen et al. present a soft tactile sensor with a 60-fold super-resolved accuracy enhanced by ANN algorithms [158]. The sensing data of neighboring units were fed into two neural networks as inputs to estimate the *x* and *y* coordinate of the load location, respectively.

Further, ML was reported to be used in the monitoring and positioning of dynamic force, which is more complicated since the transmission of the mechanical waves was induced to affect the surrounding areas of the applied impact force. Huang et al. adopted ML methods in the impact monitoring of the flexible sensing skin for the flying perception of aircraft (Fig. 13b) [99]. The output signals of a piezoelectric sensor array were collected, from which the time domain and frequency domain features were extracted to finally predict the impact position by CNN. But, regrettably, the value of impact was not estimated in this work.

### Human Posture/Motion Identification

**Fig. 14:**
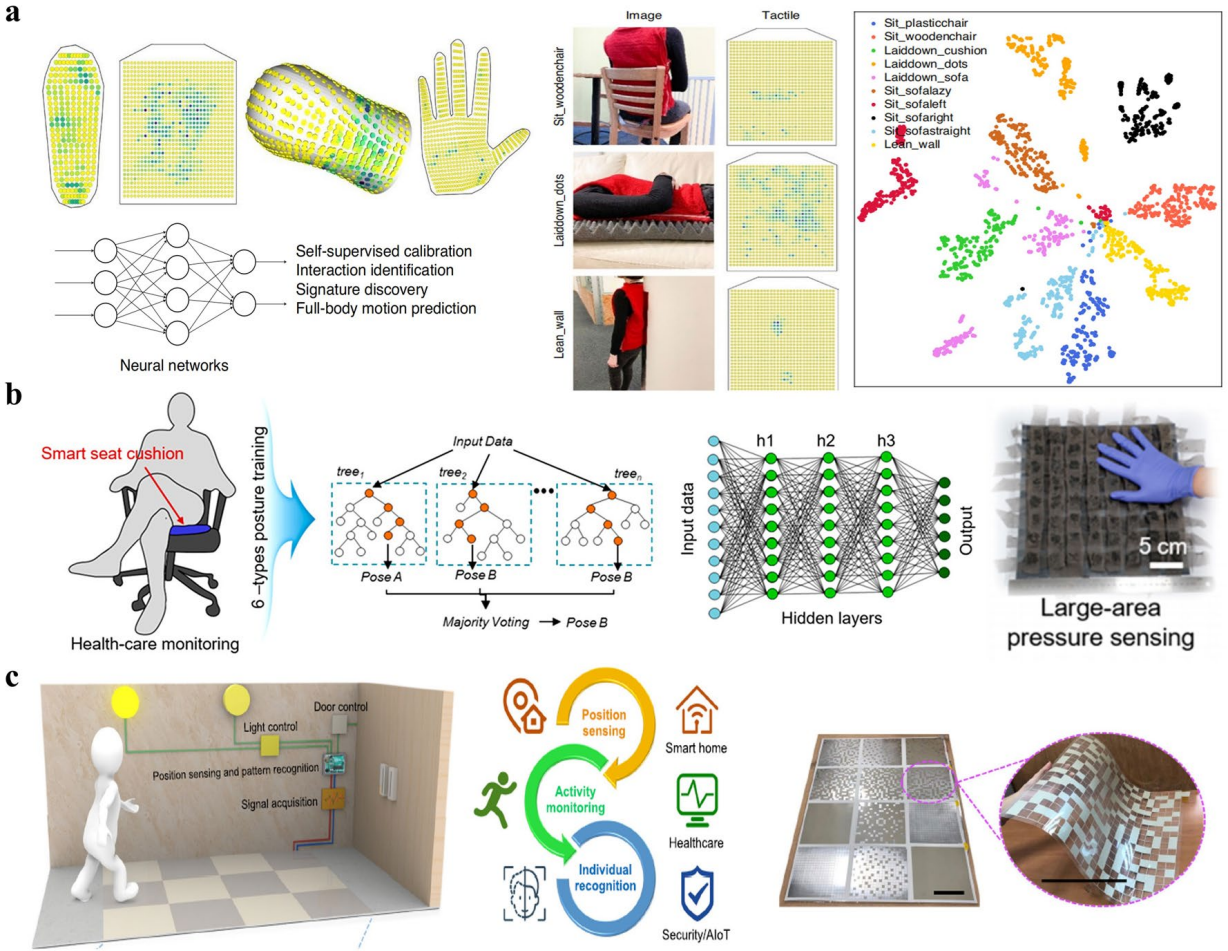
**a** Classification of human poses, motions and other interactions with the environment by conformal tactile textiles and ML techniques [187]. Copyright (2021) Springer Nature. **b** Sitting posture identification of a smart seat cushion enabled by flexible piezoresistive pressure sensor arrays and ML techniques [189]. Copyright (2021) American Chemical Society. **c** Individual recognition of a smart mat enabled by flexible triboelectric pressure sensor arrays and ML techniques [21]. (2020) The Authors

ML-assisted signal analyses have also provided various mechanical sensing arrays in wearable electronics or intelligent electric apparatus with the ability to further extract rich information about their environment and users. On one hand, the fusion of flexible sensing technology and artificial intelligence has advanced the development of wearable electronics to perceive the human body's posture and motion. Matusik et al. designed and fabricated conformal tactile textiles based on piezoresistive fiber pressure sensors (Fig. 14a) to classify humans’ sitting poses, motions, and other bodily interactions with the environment [187]. Using their sensing vest, not only different poses including sitting, standing, and reclining but also various contacted surfaces with the human body were successfully distinguished via t-distributed stochastic neighbor embedding (t-SNE). Using their sensing socks, the change of sole pressure distribution over time can be obtained to estimate the person’s pose by CNN. Similarly, Yang et al. also built several distributed insole pressure sensors of piezoresistive type to obtain the lower limb joint angles for gait phase analyses by the kNN algorithm [202]. Ferber et al. developed wearable sensors of 3D linear accelerations on the lower back, lateral thigh, and lateral shank of an individual to track subject-specific gait patterns via a one-class SVM [203]. Flexible strain sensing threads attached to the person’s neck was proposed by Sonkusale et al. to collect data for head motion classification and nine ML algorithms were compared for their application, among which linear SVM demonstrated the highest testing accuracy [204]. Differently, Fink et al. used digital temperature sensors in fibers to detect signals as inputs for a CNN, finally realizing the classification of four distinct activities including sitting, standing, walking, and running [188]. The inference was based on the fact that different human activities result in different temperature–time patterns of the human body.

On the other hand, flexible pressure sensors followed by ML-based data analysis show great promise in smart building/home applications. Tang et al. developed a smart chair with six distributed piezoresistive sensors to recognize seven different health-related sitting postures by ANN [205]. Lee et al. realized a smart seat cushion with a large-area 8 × 8 piezoresistive pressure sensor array to monitor sitting postures (Fig. 14b) using two ML methods, i.e., RF and ANN [189]. Other related works of smart chairs/cushions combining ML algorithms for human sitting posture recognition could also be found in the literature [206–209]. Similarly, Lee et al. proposed a smart toilet with a triboelectric pressure sensing array attached to its seat to achieve user identification using CNN algorithms based on different pressure distributions of individual users’ seating manner [1]. The same group also built smart mats with triboelectric pressure sensing array and utilized CNN-based data analytics for individual recognition since walking gait pattern varies from individual to individual to general unique output signal [21], as shown in Fig. 14c. It is stated that the smart floor system can still maintain a high accuracy of recognition even when the user passes through the mat array in different ways.

## Conclusion and Outlook

With the development of diversified sensing mechanisms, highly-enhanced sensing performance, more functions, and device miniaturization, the flexible electronics are fast-moving with large amounts of data and high-level features. The insufficient capacity of conventional data processing techniques to analyze big sensing data becomes apparent as it usually takes manual intervention, complicated steps, and long handling time. The flexible sensing technology can be expedited by incorporating ML methods, which can effectively deal with high-dimensional and nonlinear data to discover the intricate/hidden relationships in large datasets. In this review, we offered a glance at the recent progress in intelligent mechanical sensing technology from the combination with ML-assisted data processing algorithms. How the ML technique benefits the flexible mechanical sensing can be summarized in three aspects:

- Firstly, ML significantly improves the processing efficiency of big sensing data from large sensing arrays or/and complex sensing systems over time. The array integration of homogeneous sensing data which are measured via the same sensing mechanisms can be directly combined to derive the desired information. For example, 32 × 32 pixels of a large piezoresistive sensor array are taken as the input to a CNN for a tactile glove to identify the gripped object [34]. Further, the multimodal sensing systems with heterogeneous sensing data which are measured by different sensing mechanisms can also be comprehensively analyzed. Even visual data can be integrated with flexible strain sensors on fingers to accurately classify hand gesture, which is hard to realize without ML due to the mismatch in data dimensionality and data density [114].
- Secondly, the coupling with noise or among multiple stimuli, and overlapping among adjacent sensors, can be reduced or decoupled by ML to provide reasonable results with improved accuracy and resolution compared with those of conventional data processing techniques. For instance, the signal of a multifunctional sensor that can respond to strain, pressure, and breath stimuli is decomposed by an ET scheme to obtain every single stimulus [171]. Similarly, the common challenge to avoid device performance change induced by unwanted mechanical deformation such as bending, twisting, and stretching can also be solved by using ML for signal decomposition. Besides, bulky piezoresistive composite without array patterns can even measure the pressure value and location by inputting resistance changes at the edge into a DNN [185, 186].
- Thirdly, ML mines the hidden relationship between sensing signals and informative events. It is surprising to find that the detected ECG signal, respiration, and galvanic skin responses can collectively offer mental fatigue level information [170]. In conclusion, ML techniques have been widely proved to be a promising solution for improving the capabilities of flexible mechanical sensing without the significant update of hardware.

On the other hand, despite the rapid advances in the integration of flexible mechanical sensing with ML algorithms, the development of intelligent flexible sensing systems also faces inevitable challenges. Although ML endows the system with the ability to automatically merge all the information and learn from experience to enhance prediction accuracy, ML-assisted processing of flexible mechanical sensing data inevitably shares the pitfalls of ML algorithms. Firstly, it is often necessary to collect large amounts of diversified and rigorously vetted training data from the sensing systems to ensure a high prediction accuracy of ML model, which is a tedious and time-consuming process. For most organic material-based flexible sensors which possess intrinsic device-to-device variation and poor long-term stability, great difficulties are added in combing ML algorithms since the repeatability is directly related to model training [172]. Therefore, smarter ML algorithms need to be developed for simplified training steps and the sensor performance (especially for stability and uniformity) should be improved. Secondly, designing a proper ML model according to the sensing data and the desired outcome is a top priority. Various ML algorithms have been developed and each has merits and drawbacks to be considered in solving different situations. The hyperparameter tuning also must be conducted to find the optimal setting. For these two reasons, it should be noted that ML-assisted data processing is not always the best solution and other methods, such as linear calibrations and nonlinear fittings, could show advantages in more simplified relationships. Finally, the learning process and decision making of ML for flexible sensing data need to be regulated by corresponding knowledge and reasoning rules to make sure effective outcomes for a given application.

In the future, ML can not only be applied in the data processing of flexible sensor systems, but it would also further impact the design phase of flexible sensing systems concerning both the configuration [210] and materials [211–213]. Using inverse design realized by ML, an ideal material with desirable functionalities and an optimal sensing configuration with compact sensor implementations can be found, leading to a new generation of intelligent flexible sensing systems with powerful sensing performance [214]. Multimodal sensing platforms would be formed by integrating flexible mechanical sensing with chemical and biological sensing to provide more comprehensive information [167], where ML is expected to play an important role in both the complicated design process and data analyzing. On the other hand, in-sensor processing of flexible sensing signals by ML to provide real-time analyses is expected to be widely realized as it possesses advantages over wireless transmitting raw data to external computational devices, offering reduced communication link bandwidth and radio power requirements [24]. Personal data security can also be improved by locally processing the signals. With the continuous efforts on the improvement of sensors, microprocessor units, computing techniques, wireless communication, and AIoT [215–218], we believe that ML-enhanced flexible mechanical sensing can further improve our life quality to higher levels, ranging from health monitoring, HMI, motion/gesture identification, e-skin, to other related areas.
